# Languages with more speakers tend to be harder to (machine-)learn

**DOI:** 10.1038/s41598-023-45373-z

**Published:** 2023-10-28

**Authors:** Alexander Koplenig, Sascha Wolfer

**Affiliations:** Leibniz Institute for the German Language (IDS), Mannheim, Germany

**Keywords:** Psychology, Human behaviour

## Abstract

Computational language models (LMs), most notably exemplified by the widespread success of OpenAI's ChatGPT chatbot, show impressive performance on a wide range of linguistic tasks, thus providing cognitive science and linguistics with a computational working model to empirically study different aspects of human language. Here, we use LMs to test the hypothesis that languages with more speakers tend to be easier to learn. In two experiments, we train several LMs—ranging from very simple n-gram models to state-of-the-art deep neural networks—on written cross-linguistic corpus data covering 1293 different languages and statistically estimate learning difficulty. Using a variety of quantitative methods and machine learning techniques to account for phylogenetic relatedness and geographical proximity of languages, we show that there is robust evidence for a relationship between learning difficulty and speaker population size. However, contrary to expectations derived from previous research, our results suggest that languages with more speakers tend to be harder to learn.

## Introduction

It has long been taken for granted that there is no relationship between the structure of a language and the environment in which it is spoken^1,2^ leading to long-standing and largely unquestioned assumptions in modern linguistics that all languages are equally complex^3–11^ and equally difficult to learn^12^. Yet, depending on how you count, there are between 6000 and 8000 different languages and language varieties on the planet^13–15^ that vary widely in their structural properties^16,17^. A growing body of cross-linguistic research has begun to document that the natural and social environments in which languages are being used and learned drive this diversity^18–21^, that language structure is influenced by socio-demographic factors such as the estimated number of speakers^18,21–23^ and that the long-held belief in a principle of "invariance of language complexity"^24^ may be incorrect^25^.

In this article, we examine another long-held assumption that, to our knowledge, has never been systematically tested: the assumption that all languages are equally difficult to learn. The main obstacle to such an endeavour was already pointed out by a pioneer of modern linguistics, Henry Sweet, in 1899: “it is practically impossible for any one who has not an equally perfect knowledge of all languages to test this”^12^. In this context, cognitive scientists and computational linguists have pointed out that computational language models (LMs), most notably exemplified by the widespread success of OpenAI's ChatGPT chatbot, provide a computational working model for empirically studying various aspects of human language^26,27^. Recent research^27–30^ shows that computational models can learn core structures that are present in natural language from observed training input alone, something that was long thought to be impossible without innate linguistic knowledge^31^. In this sense, we train LMs on written text data in different languages. The LM learns to make predictions about subsequent linguistic material by finding a short encoding of the training material to which it is exposed^28,32^. With increasing input, the LM gets better at predicting subsequent data^32^. We measure how fast the LM learns to make optimal predictions and treat this as a measure of learning difficulty. We then statistically analyse this measure across different languages to test the above assumption.

Recent research using LMs in this way has indirectly suggested that languages with more speakers may be easier (i.e. faster) to learn: in a large-scale quantitative cross-linguistic analysis, Ref.^25^ trained an LM on more than 6500 documents in over 2000 different languages and statistically inferred the entropy rate of each document, which can be seen as an index of the underlying language complexity^28,33–35^. The results showed that documents in languages with more speakers tended to be more complex. Furthermore, documents that were more complex tended to be easier and faster for the LM to learn. These findings indirectly suggest that we should expect documents in languages with more speakers to be easier for the LM to learn. In this article, we first use part of the data used by Ref.^25^ to explicitly test this hypothesis. Since the LM used by Ref.^25^ is rather simple, we train two more sophisticated LMs that use machine learning and deep learning on the data and compare the results. We then discuss some potential limitations of the multilingual text collection used by Ref.^25^. To rule out that the results are driven by these limitations, we create two fully balanced and parallel multilingual corpora, which we use to train seven different LMs—ranging from very simple n-gram models to state-of-the-art deep neural networks—and measure how difficult it is for each LM to build an adequate probabilistic representation of the input.

Importantly, previous research^21,36–42^ has shown that cross-linguistic (and cross-cultural) studies that seek to analyse potential statistical associations between language features and external factors must take into account Galton's problem, which refers to the potential confounding of linguistic and cultural similarities by phylogenetic relatedness and geographical proximity. To address this issue, we take a comprehensive approach, using both established analytical methods^41^ and novel quantitative techniques developed in the field of econometrics that leverage machine learning^43,44^ and spatial autoregressive models^45^. In a series of tests, we show that there is stable evidence for an association between learning difficulty and speaker population size across LMs—but in the opposite direction to that expected from previous research, suggesting that languages with more speakers tend to be harder to (machine-)learn. We argue that this finding challenges the popular linguistic niche hypothesis^18^, which suggests that languages with larger communities of speakers should be easier, not harder, to learn.

## Results

### First study

**Figure 1:**
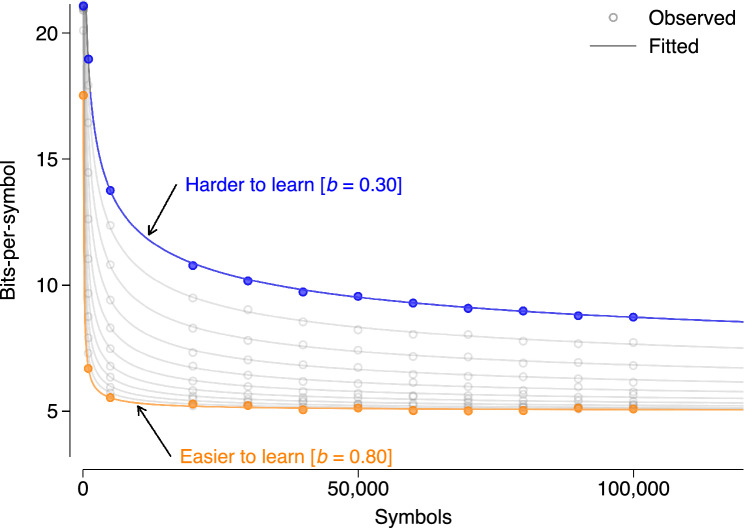
Illustration of measuring learning difficulty in Study 1. Circles represent observed bits-per-symbol that are needed (on average) to encode/predict symbols based on increasing amounts of training data for different (hypothetical) documents in different (hypothetical) languages, each with a source entropy of 5. Lines represent fitted values based on an ansatz function that has three parameters: the limiting entropy rate *h,* which quantifies how difficult it is to predict, a proportionality constant and a parameter *b*, which quantifies how difficult it is to learn to predict by describing the shape of the curve and can thus be used to quantify learning difficulty (see “Methods” for details). The blue circles illustrate a document for which learning is more difficult: to learn to make optimal predictions, the LM needs comparatively more training data and convergence to the underlying source entropy is rather slow. The orange circles, on the other hand, represent a document that is easier to learn**—**convergence is much faster: after comparatively little training data, the LM has already generated an adequate representation of the statistical structure of the input that can be used to make optimal predictions. The fitted lines show that this difference in learning difficulty can be quantified by *b*, where higher values indicate faster convergence and thus lower learning difficulty.

We first highlight some key points so readers can interpret our analyses more easily, see the “Methods” section for in-depth details. In our first series of quantitative analyses, we use part of a large-scale database of written multilingual texts comprising a variety of different text types compiled by Ref.^25^. In total, we analyse 3853 documents contained in 40 different multilingual corpora covering 1293 different languages and ranging in length from a few tens to several hundreds of millions of words. Figure 1 illustrates how learning difficulty is assessed using a shape parameter, *b*, which quantifies how difficult it is for an LM to learn to make optimal predictions^46^ (see Supplementary Fig. 1 for a further illustration). Since lower *b-*values are indicative of higher learning difficulty, we should expect a positive statistical relationship between *b* and speaker population size, if indeed languages with more speakers tend to be easier to learn.

**Figure 2:**
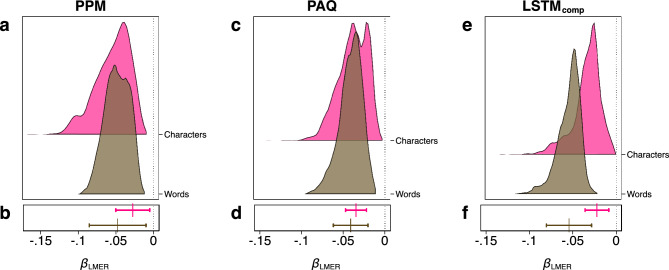
Multilevel mixed-effects linear regression results (Study 1) for three different LMs—PPM, PAQ and LSTM_comp_. (**a**), (**c**) and (**e**). Distribution of the estimated impact of speaker population size, *β*_LMER_, per LM and per symbol for a total of 2430 models that include a fixed effect (and potential random slopes) for speaker population size. To control for the non-independence of data points due to phylogenetic relatedness and geographic proximity, all models additionally include fixed covariates, random intercepts and random slopes (see “Methods” for details). (**b**), (**d**) and (**f**). Estimated *β*_LMER_ (vertical line) and 95% confidence interval (horizontal line) for the model with the lowest AIC per LM and per symbol (see Supplementary Table 1 for numerical results and model specifications). Olive colour—words as information encoding units. Pink colour—characters as information encoding units.

We first focus on prediction by partial matching (PPM)^47^, which is based on a variable-order Markov LM (see “Methods” for details). To this end, we use estimates for *b* measured for both words and characters as information encoding units provided by Ref.^25^. On both levels (words/characters), we run separate multilevel mixed-effects linear regressions (LMER) with *b* as the outcome. We include fixed effects for the (log of) speaker population size and, to account for document- and corpus-specific characteristics, the entropy rate *h*, the text length *L* and the interaction between *h* and *L*. To account for the potential non-independence of data points described above, we include (crossed) random intercepts for the following groups: corpus, language family, language, macro area, country and writing script. In addition, we include random slopes (i.e. we allow the effect of population size to vary across different groups) for all groups except language (since population size does not vary within languages). Given the absence of clear theoretical or empirical reasons to determine which covariates to include, we adopted a multi-model inference approach^48^ by subsetting the full model, i.e. we generated a set of models with all possible covariate subsets, which were then fitted to the data. In total, we ran 4860 different sub-models (see “Methods” for details). As a means of selecting between models, we use Akaike’s information criterion (AIC)^49^ where lower values indicate a more apt model. A comparison of reduced models without a fixed effect (and potential random slopes) for speaker population size with full models where speaker population size is included reveal that in all 2430 possible model pairs, for both words and characters, the model that includes speaker population size has a lower AIC (median difference between reduced and full models ΔAIC_med_ = 46.55 for words and ΔAIC_med_ = 71.16 for characters, see Supplementary Table 1 for numerical results). This result clearly points towards a statistical association between learning speed and population size. However, Fig. 2a shows that all *β*-coefficients, *β*_LMER_, estimated for speaker population size are negative for both words and characters indicating that larger population sizes are associated with lower values of *b* and thus higher learning difficulty. To account for the uncertainty in the model selection process, we compute a frequentist model averaging (FMA) estimator^50^ (see “Methods” for details), ${\beta }_{LMER}^{FMA}$ = − 0.050 for words and ${\beta }_{LMER}^{FMA}$ = − 0.030 for characters. This indicates that languages with more speakers tend to be harder for PPM to learn. Figure 2b shows the estimated *β*-coefficients and 95% confidence intervals for the best models, i.e. the models with the lowest AIC for both symbolic levels. In both cases, an increase in speaker population size predicts a decrease in *b* and thus higher learning difficulty (both parametric *p*-values < 0.05).

To test whether these results are specific to PPM, whose LM is relatively simple^25^, we trained two further LMs on all written data from Ref.^25^: (i) PAQ^51^, which employs several machine learning techniques for prediction^52^ and (ii) LSTM_comp_^53^, which uses a deep learning model^54^ for prediction (see “Methods” for details).

The results obtained from these algorithms strongly support the results obtained from PPM. For PAQ, all models that include speaker population size as a covariate have a lower AIC than reduced models for words (ΔAIC_med_ = 36.87) and 2422 out of all 2430 full models have a lower AIC than reduced models (99.67%) for characters (ΔAIC_med_ = 37.76). For LSTM_comp_, all models that include speaker population size as a covariate have a lower AIC than reduced models for both words and characters (ΔAIC_med_ = 62.81 for characters and ΔAIC_med_ = 54.84 for words). Figure 2c,e shows that for both algorithms, the estimated *β*-coefficients for speaker population size were consistently negative for both characters and words in all models. For PAQ, ${\beta }_{LMER}^{FMA}$ = − 0.043 for words and ${\beta }_{LMER}^{FMA}$ = − 0.035 for characters. For LSTM_comp_, ${\beta }_{LMER}^{FMA}$ = − 0.059 for words and ${\beta }_{LMER}^{FMA}$ = − 0.023 for characters. Figure 2d,f visualises the estimated *β*-coefficients and 95% confidence intervals for the best models per symbol. For both LMs and on both levels (words/characters), the confidence intervals do not include zero. Supplementary Table 1 provides numerical details and shows that all four estimates are statistically significant at *p* < 0.005. In Supplementary Table 2, we show that the results hold when documents from comparable corpora are excluded and only fully parallel corpora are considered (see “Methods” for details).

To further estimate the potential effect of speaker population size on learning difficulty while controlling for potential confounding due to translation effects^55^ and pluricentism^56^ in addition to covariation due to phylogenetic relatedness and geographic proximity, we generated three sets of potential control variables. The small set, consisting of a total of *N*_c_ = 225 candidates, includes these variables:

(i) A set of indicator variables for the levels (categories) of corpus, language family, writing script, macro area and the Expanded Graded Intergenerational Disruption Scale (EGIDS)^57^;
(ii) To control for geographical proximity, third-order B-spline basis functions for both latitude and longitude^43,58^;
(iii) Several other continuous environmental variables in addition to *h* and *L*, such as the number of countries in which a language is spoken, geographical range, altitude and climate (see “Methods” for details).

In addition to the variables of the small set, the medium set (*N*_c_ = 274) includes first order/two-way interactions of the basis functions of (ii). In addition to the variables of the medium set, the big set (*N*_c_ = 2226) includes first-order interactions between the indicators of (i).

To estimate the effect of speaker population size on learning difficulty in such a high-dimensional setting, we use a technique called double selection^43^, which uses the lasso machine learning technique^59^ to select the relevant control variables from the candidate set (see “Methods” for details).

**Figure 3:**
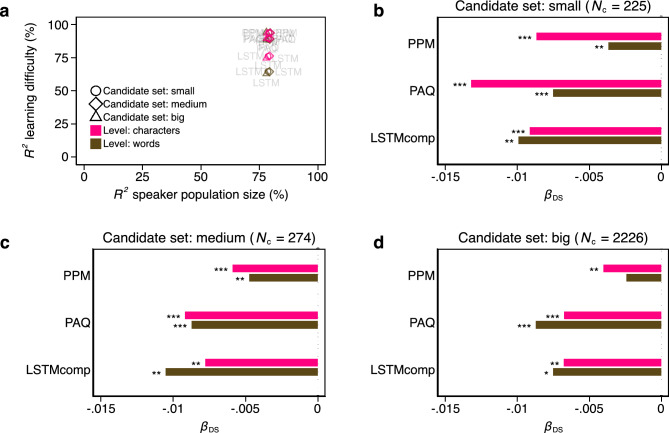
Double-selection lasso linear regression results (Study 1). (**a**) Prediction performance: out-of-sample *R*^2^ of learning difficulty against Out-of-sample *R*^2^ of speaker population size. In both cases, the out-of-sample *R*^2^ is computed for a sample distinct from the sample for which the control variables were selected by the lasso for the three different candidate sets. (**b**), (**c**) and (**d**) Bars—estimated coefficients, *β*_DS_, for the effect of speaker population size per LM and per symbol. (**b**) Candidate set: small (number of control covariates *N*_c_ = 225), (**c**) candidate set: medium (*N*_c_ = 274), (**d**) candidate set: big (*N*_c_ = 2226). *p < 0.05, **p < 0.01, ***p < 0.005. Statistical significance is determined based on non-parametric permutation tests (see “Methods” for details and Supplementary Table 3 for numerical results). Olive colour—words as information encoding units. Pink colour—characters as information encoding units.

Figure 3a shows that all selected models have high predictive power^60^ explaining between 62.90% and 94.47% of the total variance in learning difficulty (median out-of-sample ${R}_{OS}^{2}$ = 89.23%) and between 77.95% and 79.68% of the total variance of speaker population size (median ${R}_{OS}^{2}$ = 78.82%). Note that the use of standard parametric tests may be questioned in this study, as the sample of languages for which we have available documents cannot be considered a random sample of the population of all languages^61,62^. To address this issue, we used the selected relevant controls as input for Freedman-Lane permutation tests^63^ to compute non-parametric *p*-values (see “Methods” for details).

Figure 3b–d shows that the *β*-coefficient for speaker population, *β*_DS_, size remains negative in all scenarios and passes the permutation test at *p* < 0.05 in all but one case. The exception is PPM on the level of words for the big candidate set. Supplementary Table 3, which contains numerical results, shows that in this case, *p* = 0.07. In Supplementary Table 4, we show that the results also hold when only fully parallel corpora are considered. Again, *β*_DS_ remains negative in all scenarios and passes the permutation test at *p* < 0.05 in all but one case. To further assess the robustness of these findings, we employ a more computationally intensive technique known as cross-fit partialing-out (or double machine learning)^44^ to estimate the effect of speaker population size on learning difficulty and compute parametric *p*-values. This method has a less restrictive sparsity requirement and provides an additional validation of our results: Supplementary Table 5 shows that that the *β*-coefficient for speaker population is again negative and significant (at *p* < 0.005) in all cases. In Supplementary Table 6, we adopt a Bayesian perspective by using the lasso-selected controls as input for Bayesian linear regression models and show that, consistent with the results presented here, the probability of the coefficient of speaker population size being negative was estimated to be 1 across all compressors and both symbolic levels.

### Second study

While the results presented in the previous section indicate that texts in languages with more speakers tend to be harder to learn, it is important to rule out that the results are mainly driven by several limitations inherent in the multilingual corpus collection used^25^: First, most of the texts in the database are rather short (median length = 150,188 words; first quartile *Q*_1_ = 3385; third quartile *Q*_3_ = 234,838). This can be a problem as LMs, especially more complex ones, typically require a lot of training input in order to achieve good performance^27,64^. Secondly, learning difficulty as defined by Ref.^46^ ultimately rests on an ansatz function that cannot be proven analytically^65^. Thirdly, the database is unbalanced at the language level: while there are more than 100 languages with at least 10 available data points, i.e. training documents, there are less than four available data points for most languages (~ 84%). This reflects the fact that for languages spoken by a small number of people, there are only very few documents available electronically^66^. This unbalancedness precludes the use of several approaches that have been discussed and successfully used in the literature^41^, but require balanced data as input.

**Figure 4:**
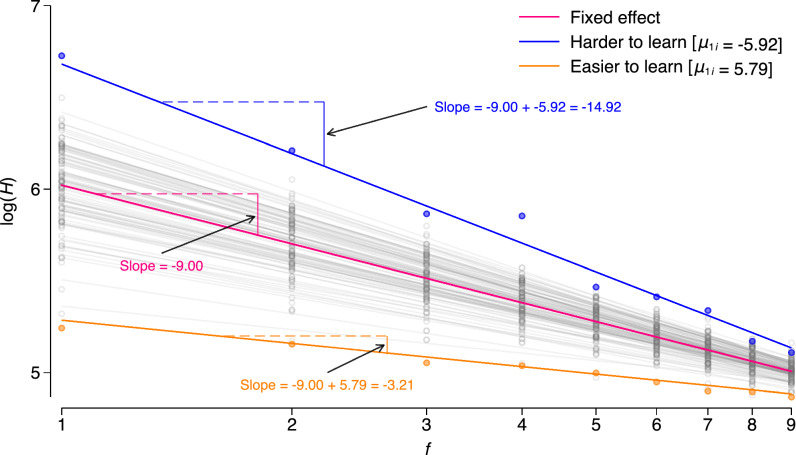
Illustration of measuring learning difficulty in Study 2. Circles represent observed cross entropies *H* that are required (on average) to encode/predict the test data as a function of the number of training folds, *f*. We fit an LMER with the log of *f* as a fixed effect and random intercepts for the test fold and language. We include random slopes per language, which are represented by the different lines in the figure. The pink line corresponds to the estimated fixed effect of *f*, here a value of − 9.00. Using the LMER, we obtained language-specific best linear unbiased predictions (BLUPs) of the random slopes, represented by the value of *µ* for each language. These BLUPs capture the interactions between *f* and language, with higher values of *µ* indicating faster learning and lower values indicating slower learning. As illustrated by the orange circles/line in the figure, some languages have higher values of *µ*, indicating that they are easier to learn. For these languages, the LM achieves better prediction quality with fewer training data points, resulting in a flatter slope of the regression line. The blue circles/line, on the other hand, represent languages that are more difficult to learn. These languages require more training data for the LM to achieve better levels of prediction quality, resulting in steeper slopes of the regression line.

In consideration of these issues, we used the Parallel Bible Corpus^67^, which is available in a very fine-grained parallel structure (in terms of book, chapter and verse) and that provides additional information regarding the genealogical classification of languages. We created two fully balanced and parallel multilingual training corpora: (i) a New Testament (NT) version consisting of 5000 parallel verses available in 504 different languages and (ii) an Old Testament (OT) version consisting of 15,000 parallel verses available in 138 different languages. While the “Methods” section provides detailed information, we again would like to highlight a few key points here to facilitate the interpretation of our analyses: per version (NT/OT), we randomly assigned each available verse to one of ten folds. Per language, we then conducted a tenfold rotation estimation where each fold served once as the test data and the remaining folds were used as training data resulting in 9 (data points per rotation) × 10 (folds) = 90 data points per version and language. For example, if fold 10 serves as the test data, we will train an LM on one of the remaining folds (randomly selected). We then computed the cross entropy *H*, i.e. the average number of bits per verse required to encode/predict each byte of fold 10 as a measure of the quality of the language model^68^. Next, we added another of the remaining folds to the training data, re-trained the LM and calculated *H* again. This process was repeated until all nine folds have been used as training data. Per version, we then use all the resulting data points to fit an LMER with *H* as the outcome and a fixed effect for *f*, the number of folds used for training. We include (crossed) random intercepts for the test fold and for language. Crucially, we include random slopes per language, i.e. we allow the relationship between *H* and *f* to be different for each language. As illustrated in Fig. 4, this random slope can be used as a measure of learning difficulty: the slope measures how much additional input improves the quality of the LM—for a language that is easy to learn, the LM has already generated an adequate representation of the input after the first (few) folds resulting in a slope that is comparatively less steep. For a language that is difficult to learn, the LM needs more input to learn to predict. The LM should therefore improve its quality more with more input, resulting in a comparatively steeper slope. In other words, the random slope parameter we are analysing here modulates the general relationship between *H* and *f* for all languages. The language-specific value of this random slope parameter is then indicative of a stronger or weaker relationship between *H* and *f* for that language.

As information encoding units, we estimate on two levels: on the level of words and, instead of estimating on the level of characters, we tokenize our text into sub-word units by byte pair encoding (BPE)^69,70^ which plays an important role in many state-of-the-art natural language model applications^71,72^ and provides strong baseline results on a multilingual corpus^73^. In total, we trained seven different LMs on the data—ranging from very simple n-gram models to state-of-the-art deep neural networks (Table 1).

**Table 1:** Language models used in Study 2.

LM	Technique/algorithm	Source	Time
PPM2	N-gram modelling^74^, prediction by partial matching^47^, number of previous symbols: 2, memory: 2000 megabytes	Ref.^75,76^	0.1
PPM6	N-gram modelling^74^, prediction by partial matching^47^, number of previous symbols: 6, memory: 2000 megabytes		0.1
LZMA	Dictionary encoding^77^, dictionary size 1536 megabytes	Ref.^76^	0.2
PAQ	Context mixing^52,78^, gated linear network^79^, ~ 1.7 million weights, parameters ~ 3800	Ref.^51,80^	60.7
LSTM_comp_	Long short term memory^54^, parameters ~ 0.5 million	Ref.^53^	149.2
NNCP_small_	Transformer^64^, parameters ~ 2.24 million	Ref.^81^	146.0
NNCP_large_	Transformer^64^, parameters ~ 6.45 million		431.9

The language models used in Study 2 are listed along with their implementation techniques, source, and time (in seconds) required to train each model on a median length document. The first three models are relatively simple, while the remaining four are more complex. The first four models were trained on 231,480 documents while the last three were trained on 115,740 documents due to the significant increase in training time, i.e. we only used the first five folds as test folds for the last three LMs, resulting in 45 data points per version and language, whereas all ten folds were used as test folds for the first four LMs, resulting in 90 data points per version and language. Further details on training and implementation are provided in the “Methods” section.

**Figure 5:**
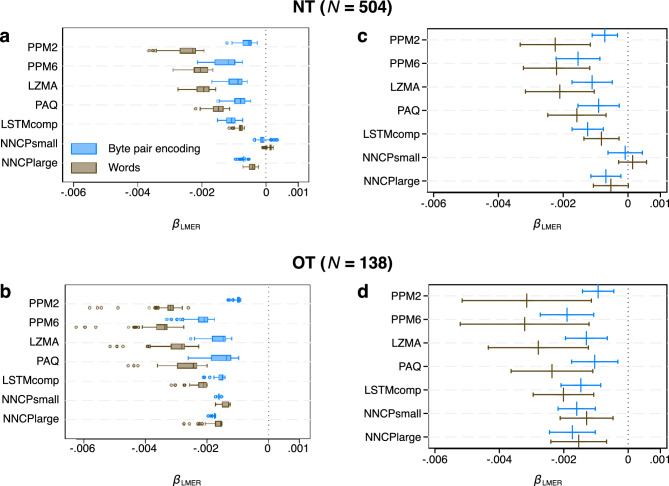
Multilevel mixed-effects linear regression results (Study 2). (**a**) and (**c**) Results for NT (*N* = 504). (**b**) and (**d**) Results for OT (*N* = 138). (**a**) and (**b**) Boxplots visualising the distribution of the estimated impact of speaker population size, *β*_LMER_, per LM and per symbol for a total of 2430 models that include a fixed effect (and potential random slopes) for speaker population size. (**c**) and (**d**) Estimated *β*_LMER_ (vertical line) and 95% confidence interval (horizontal line) for the model with the lowest AIC per LM and per symbol (see Supplementary Table 7 for numerical results and model specifications). Olive colour—words as information encoding units. Blue colour—byte-pair encoding.

Per LM, per version (NT/OT) and per symbolic level (words/BPE), we estimated language-specific random slopes, which serve as measures of learning difficulty as shown in Fig. 4. To test for a potential relationship between population size and learning difficulty, we first ran separate LMERs with learning difficulty, *µ*, as the outcome. Analogous to Study 1, we created a maximum model that contains a fixed effect of the log of speaker population size and (crossed) random effects and slopes for writing script, macro area, country, language family, language subfamily and sub-branch. We then computed LMERs for all possible covariate subsets (1456 models). Figure 5a,b shows that with the exception of the small transformer model for the NT version, all computed FMA estimates for all *N* = 728 models that include a fixed effect for speaker population size (and potential random slopes) are negative for both corpus versions and both symbolic levels. For the NT version, ${\beta }_{LMER}^{FMA}$ = − 0.0022 for words and ${\beta }_{LMER}^{FMA}$ = − 0.0007 for BPE for PPM2 as LM, ${\beta }_{LMER}^{FMA}$ = − 0.0019 for words and ${\beta }_{LMER}^{FMA}$ = − 0.0016 for BPE for PPM6, ${\beta }_{LMER}^{FMA}$ = − 0.0019 for words and ${\beta }_{LMER}^{FMA}$ = − 0.0011 for BPE for LZMA, ${\beta }_{LMER}^{FMA}$ = − 0.0014 for words and ${\beta }_{LMER}^{FMA}$ = − 0.0009 for BPE for PAQ, ${\beta }_{LMER}^{FMA}$ = − 0.0008 for words and ${\beta }_{LMER}^{FMA}$ = − 0.0011 for BPE for LSTM_comp_, ${\beta }_{LMER}^{FMA}$ = − 0.0002 for words and ${\beta }_{LMER}^{FMA}$ = − 0.0001 for BPE for NNCP_small_ and ${\beta }_{LMER}^{FMA}$ = − 0.0005 for words and ${\beta }_{LMER}^{FMA}$ = − 0.0007 for BPE for NNCP_small_. For the OT version, ${\beta }_{LMER}^{FMA}$ = − 0.0032 for words and ${\beta }_{LMER}^{FMA}$ = − 0.0010 for BPE for PPM2, ${\beta }_{LMER}^{FMA}$ = − 0.0033 for words and ${\beta }_{LMER}^{FMA}$ = − 0.0020 for BPE for PPM6, ${\beta }_{LMER}^{FMA}$ = − 0.0028 for words and ${\beta }_{LMER}^{FMA}$ = − 0.0014 for BPE for LZMA, ${\beta }_{LMER}^{FMA}$ = − 0.0024 for words and ${\beta }_{LMER}^{FMA}$ = − 0.0011 for BPE for PAQ, ${\beta }_{LMER}^{FMA}$ = − 0.0021 for words and ${\beta }_{LMER}^{FMA}$ = − 0.0015 for BPE for LSTM_comp_, ${\beta }_{LMER}^{FMA}$ = − 0.0013 for words and ${\beta }_{LMER}^{FMA}$ = − 0.0016 for BPE for NNCP_small_ and ${\beta }_{LMER}^{FMA}$ = − 0.0015 for words and ${\beta }_{LMER}^{FMA}$ = − 0.0017 for BPE for NNCP_small_. Figure 5c,d visualizes the estimated *β*-coefficients and 95% confidence intervals for the models with the lowest AIC per version, LM and symbolic level. There is a significant negative impact of population size on learning difficulty (*p* < 0.005) for all language models in the NT version, except for the two transformer models (see Supplementary Table 7 for numerical results and model specifications). Here, only the coefficient for the larger transformer on the BPE level was statistically significant (*p* < 0.005), while both coefficients for the small transformer LM were deemed non-significant. Given that transformers are known to require large amounts of training data to perform well^64^, we attribute the lack of significance in the coefficients based on the smaller transformer to the limited size of the training data used for the NT version. This assumption is consistent with the fact that all *β*_LMER_-values at both symbolic levels were negative at *p* < 0.005 for the OT version, where the amount of training data is three times larger. Supplementary Table 7 shows that these results are corroborated by the ΔAIC-values. In addition, we show in Supplementary Table 8 that these results are fully supported by lasso linear regressions similar to those presented in Supplementary Tables 3, 4, 5.

**Figure 6:**
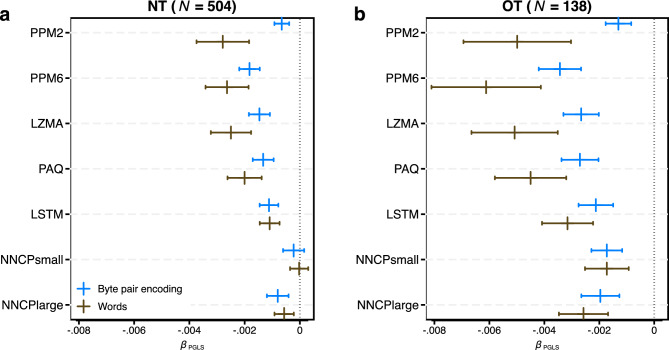
Phylogenetic generalized least squares regression results (Study 2). Estimated *β*_PGLS_ (vertical line) and 95% confidence interval (horizontal line) per LM and per symbol (see Supplementary Table 9 for numerical results). (**a**) Results for NT (N = 504). (**b**) Results for OT (N = 138). Olive colour—words as information encoding units. Blue colour—byte-pair encoding.

To further investigate the relationship between language learning difficulty and population size, we proceed by explicitly modelling the degree of covariation due to descent from a common ancestor. To this end, we conduct a Phylogenetic Generalised Least Squares (PGLS) regression with learning difficulty as the outcome and speaker population size as a covariate^41^. We use a phylogenetic tree provided by Ref.^82^ that was generated using language taxonomies from Ethnologue^83^. Fig. 6 presents the results, which are in close agreement with the LMER results (Fig. 5c,d): with the exception of the small transformer model for the NT version, all *β*_PGLS_-coefficients are negative at *p* < 0.005 (see Supplementary Table 9 for numerical details). The information-theoretic approach where we calculate ΔAIC between reduced models that do not include population size and full models, again supports these results. Excluding the small transformer model for the NT version, speaker population size explains the median amount of variance in learning difficulty of ${R}_{med}^{2}$ = 7.59% (Q_1_ = 5.29%, Q_3_ = 8.42%). For the OT version, the results for all LMs are more pronounced (${R}_{med}^{2}$ = 22.00%, Q_1_ = 18.49%, Q_3_ = 25.33%, no exclusion of the small transformer model).

While the PGLS analyses explicitly control for genealogical relatedness, spatial proximity can also generate non-independence in comparative language data^21,41,84^. To control for both sources of influence simultaneously, we use two weighting matrices:

(i) to control for spatial proximity, we generate a matrix containing the geographical distances between languages;
(ii) to control for genealogical relatedness, we used a phylogenetic dissimilarity matrix provided by Ref.^85^ that is based on word lists from the Automated Similarity Judgment Program (ASJP)^86^.

We conduct spatial autoregressive errors regression models (SAR)^45^ with learning difficulty as the outcome and speaker population size as a covariate. We add two spatially lagged error terms specified by the inverse of each weighting matrix using a generalized spatial two-stage least-squares estimator (GS2SLS)^87^.

**Figure 7:**
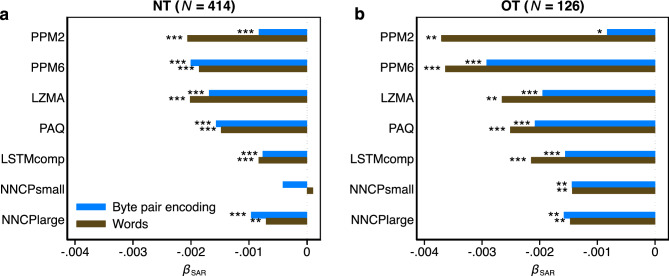
Spatial autoregressive error regression results (Study 2). Bars—estimated coefficients, *β*_SAR_, for the effect of speaker population size per LM and per symbol. (**a**) Results for NT (N = 414). (**b**) Results for OT (*N* = 126). Each model contains autoregressive error terms for phylogenetic relatedness and geographical proximity simultaneously estimated by two inverse-distance matrices. *p < 0.05, ** *p* < 0.01, *** *p* < 0.005. Statistical significance is determined based on non-parametric permutation tests (see “Methods” for details and Supplementary Table 10 for numerical results). Olive colour—words as information encoding units. Blue colour—byte-pair encoding.

The SAR results are visualized in Fig. 7. Again, all results are in close agreement with the other analyses presented in this section: 27 out of 28 estimated *β*_SAR_-coefficients are negative. With the exception of the small transformer model for the NT version, all coefficients pass a non-parametric permutation test (see “Methods” for details) with *p* < 0.05 in one case, *p* < 0.01 in four cases and *p* < 0.005 in the remaining 21 cases (goodness-of-fit: NT version, excluding NNCP_small_: ${R}_{med}^{2}$ = 8.64%, Q_1_ = 5.57%, Q_3_ = 10.44%; OT version: ${R}_{med}^{2}$ = 17.02%, Q_1_ = 14.32%, Q_3_ = 20.38%, see Supplementary Table 10 for numerical details).

## Discussion

In this article, we examined the assumption that all languages are equally difficult to learn by using LMs as computational working models for empirically studying various aspects of human language^26^. In summary, we find that there is evidence for an effect of speaker population size on learning difficulty that questions the above assumption. This evidence turns out to be stable across different datasets and two different ways of operationalising learning difficulty. We have observed this relationship across a range of LMs, ranging from very basic n-gram models that use only the last few symbols for prediction to state-of-the-art deep neural network large language models that leverage complex computational architectures and mechanisms that allow them to capture long-range dependencies and contextual information in the text. Despite the differences in model complexity, the observed correlation held consistently, highlighting the robustness of this finding across the spectrum of LMs.

To address the potential non-independence of observations resulting from the phylogenetic and geographic relationships between languages, we employed established methods from existing literature^41^ while also introducing novel approaches to analyse our data^43,45^. In addition, we used both parametric and non-parametric tests to determine statistical significance, recognising that our data cannot be considered a random sample of all existing languages^61^.

By using a variety of methods and analyses, we have increased the reliability and generalisability of our primary finding, which challenges our initial expectations: contrary to what we expected, our research reveals a positive statistical association between population size and learning difficulty, suggesting that languages with more speakers tend to be harder to learn.

The expectation that there should be an inverse correlation between speaker population size and learning difficulty can be traced back to the linguistic niche hypothesis, which suggests that the social niche that a language occupies in a community affects its structural properties^2,18^. Specifically, the linguistic niche hypothesis suggests that languages with large numbers of speakers tend to simplify their grammar and have a reduced structural complexity. It assumes that languages that are spoken by more people over larger geographical areas are, on average, also learned by a larger proportion of adults. Since languages with complex structures appear to be difficult for adults to learn, the linguistic niche hypothesis conjectures that there should be a negative selection against complexity, i.e. languages tend to adapt and simplify when they are spoken by larger communities that include a significant number of adult learners^18,88,89^. Subsequently, the linguistic niche hypothesis has been an important starting point that generated extensive research in the field^89–95^. Only very recently, research has emerged that casts doubt on the validity of the specific assertions made by the linguistic niche hypothesis: it has been shown that the number of adult learners does not appear to impact language complexity^21,23^ and that languages with more speakers tend to be more complex, not less^21,25^. In a similar vein, the results presented in this article suggest that languages with more speakers are not easier to learn but more difficult.

It is important to point out in this context that LMs are only working models and that there are therefore important limitations^26^. In particular, we neither claim that there is a one-to-one correspondence between human and machine language learning, nor that LMs understand language in a human-like sense^96^. Future work could explore whether and to what extent our results also apply to human language learning. However, we agree with Refs.^26,27^ that, given their impressive performance in natural language processing^71,97^, LMs, especially so-called large language models, are worthy of scientific investigation, because of their inherent “potential to inform future work in the cognitive science of language”^26^. In our study we have tried to exploit this potential, and we hope that we have, at a very minimum, been able to show that the sociolinguistic structure of language and learnability by machines do not seem to be statistically independent of each other.

Nevertheless, as language scientists, our primary objective is to comprehend the underlying reasons behind the specific characteristics of human language, and it is not clear whether the results regarding machine learnability presented in this study can be extrapolated to human language learning as well. A link to human language learning could be made through the field of artificial grammar learning, where participants are asked to judge the permissibility of an upcoming symbol in a sequence of symbols based on the rules of an underlying artificial grammar. In this paradigm, it can be shown that the complexity of an artificial grammar is positively correlated with the error rate of participants who implicitly learn the underlying rules of an artificial grammar (i.e., by reading language material based on the artificial grammar)^98,99^. Our results suggest that rule complexity should also influence the speed of artificial grammar learning (measured, e.g., as number of experimental trials until the rules have been extracted from the linguistic material).

Using advanced machine learning techniques, we found that speaker population size negatively affects learning speed after controlling for potential confounding from translation effects and several environmental variables such as geographic range size. To understand how speaker population size affects machine learning difficulty, future studies could build on this methodological approach and examine the impact of other potential covariates. Another promising avenue for future work would therefore be to investigate which types of grammatical structures tend to be more difficult for LMs to learn, and whether those features covary with speaker population size. A recently published new cross-linguistic database of grammatical features of unprecedented size^17^ provides an ideal starting point for such endeavours.

## Methods

### Language models

We use general-purpose data compression algorithms, taking advantage of the fact that there is a close connection between understanding, prediction and compression^100,101^. All data compression algorithms consist of a model and a coder^78^. Our focus is on the class of (lossless) compressors where the algorithm estimates a model, i.e. a conditional probability distribution, based on the training data, which can be used to generate predictions. To perform compression, the predicted probabilities are then used to encode symbols using a technique called arithmetic encoding^102^. The language models that we use are summarized in Table 1. In what follows, further details are given for each language model. *PPM* is a dynamic and adaptive variable-order n-gram LM. The algorithm makes an assumption of the Markov property: to predict the next symbol, the algorithm uses the last *o* symbols that immediately precede the symbol of interest^47,103^. For Study 2, we use two different values for *o*, 2 and 6, i.e. the last 2 resp. 6 symbols are used as context to generate predictions, whereas the optimal order in the range of [2, 32] is learned directly from the data in Study 1^25,46^. In both studies, the level of compression is set to maximum and the size of used memory is set to 2000 megabytes. *LZMA* employs a compression strategy wherein repetitive segments within the data are identified and replaced by references pointing to a single instance of that segment occurring earlier in the uncompressed data stream. These matches are encoded using a length-distance pair, indicating that a specific number of symbols following the match are identical to the symbols located a certain distance back in the uncompressed stream^77,78^. LZMA is only used in Study 2; the level of compression is set to maximum and the size of the compression dictionary is set to the maximum value of 1536 megabytes. *PAQ* can be described as a weighted combination of predictions from a large number of models, where the individual models are combined using a gated linear network^52,78–80^. The network has a single layer with 552 input nodes and 3080 input weights. The model has a total of approximately 1.7 million weights, but due to the sparse updating scheme which leads to faster compression and decompression, the effective number of parameters used in training is significantly lower. Only 552∙7 = 3864 weights are updated for each bit of data. In both studies, we use version PAQ8o and set the compression level to maximum, requiring 1712 megabytes of memory. As PAQ, lstm-compress^53^, referred to as *LSTM*_*comp*_ throughout the manuscript, combines predictions from independent models. Predictions are combined using a long short-term memory deep neural network^54^. The network is trained using backpropagation through time^104^ and Adam optimisation is used to update network weights^105^. The algorithm takes no options and we do not use any dictionary-based pre-processor in neither Study 1 nor Study 2. In total, the model has 508,936 parameters (assuming the corresponding input file uses all 256 possible bytes. The model size may be smaller if the input file contains a smaller set of bytes). NNCP^81^ is a lossless data compressor that is based on the Transformer XL model defined in Ref.^97^. Modifications to the original Transformer XL model and algorithmic details are provided in Refs.^106,107^. As for lstm-compress, the Adam optimiser is used. For *NNCP*_*small*_, we use the default options with four layers and a resulting total number of parameters of ~ 2.24 million. For *NNCP*_*large*_, we change the default options to twelve layers. This results in a total of ~ 6.45 million parameters. For both versions, we use the Gaussian error linear unit activation function^108^, we do not use a text pre-processor or tokenizer and we use the faster “encode only” mode (the output cannot be decompressed, but the compression itself is still lossless). NNCP is only used in Study 2.

### Data

For *Study 1*, we use part of a large-scale database of written multilingual texts compiled by Ref.^25^. In total, we use information on 3853 documents contained in 40 different multilingual corpora comprising a large variety of different text types. The documents range in length from a few tens to several hundreds of millions of words. 33 of these corpora consist of fully parallel texts. Parallel texts are texts in different languages that contain the same message, but differ in the language used (e.g. subtitles of a movie in different languages). The remaining seven corpora are comparable corpora, i.e. texts that are not parallel but come from comparable sources and are therefore similar in content (e.g. Wikipedia or newspaper articles). In-depth details on the database and each corpus are given in the “Methods” section and supplementary information of Ref.^25^ that is available at https://osf.io/f5mke/.

For *Study 2*, we use data from the Parallel Bible Corpus made available by Ref.^67^, which contains 1568 unique translations of the Bible in 1166 different languages in a fine-grained parallel structure (in terms of book, chapter and verse). Each translation is already tokenized and Unicode normalized and spaces are inserted between words as well as punctuation marks and non-alphabetic symbols by Ref.^67^. In addition, all texts were manually checked and corrected by Ref.^67^ where necessary. In some texts without spaces or marks between words (e.g. for Khmer, Burmese, or Mandarin Chinese), we used a dictionary lookup method described in Ref.^25^ to detect word boundaries with detected word tokens then being space-separated. All uppercase characters are lowered based on the closest language-specific ISO-639-3 code. We then split each Bible translation into different books of the biblical canon, aggregating all books of the New Testament (NT) and the Old Testament (OT). Beside the actual text, each element of each line of each Bible translation document contains information about the book, chapter and verse number^67^. Per version, we dropped translations with no available verse. For languages with more than one available translation, we kept the translation with most available verses and broke ties at random. In total, we ended up with 1062 different languages for the NT version and 189 different languages for the OT version. On average, each NT translation consists of 7840 verses and each OT translation consists of 17,086 verses. Per version, we then dropped partly incomplete translations and removed verses that are only available in some translations and selected verses that appeared in as many translations as possible. For the NT version, we selected 5000 verses that are available in 504 different languages. For the OT version, we selected 15,000 parallel verses in 138 different languages. Note that the biblical canon consists of different books, for our selection 24 books for NT and 36 books for OT. Per version (NT/OT) we prepared stratified samples by randomly assigning each available verse from each available book to one of ten folds. In addition, we made sure that both (i) the verse order across translations and folds and (ii) the sequential training order is fully balanced and parallel; (i) means that each fold consists of the same verses that the LM is trained with, in the same order. From an information-theoretic perspective, this procedure ensures that—apart from random fluctuations—each fold contains text drawn from the same information source^109^ and thus induces stationarity^110^. Regarding (ii), assume that fold 10 is the test fold, and the remaining folds are used to sequentially train the LM. We generated random training sequences, e.g. fold 3–fold 2–fold 8–fold 5–fold 4–fold 9–fold 6–fold 7–fold 1. This means that the LM is first trained on fold 3, we then compute *H*, i.e. the average number of bits per verse that are needed to encode each byte of fold 10, then the LM is trained on fold 3 and fold 2 and *H* is computed again, and so on. Training sequences are kept parallel across translations.

### Information encoding units

For *Study 1*, we follow Ref.^25^ and compute the relevant quantities for both words and characters as information encoding units/symbols. For *Study 2*, we estimate on the level of words, but not on the level of characters, since there are idiosyncrasies/vagaries of the writing system that can lead to cross-linguistic differences in the mapping between phonemes and graphemes on the level of characters^111,112^. Instead, we apply byte pair encoding (BPE)^69,70^ to split words into one or several units and the LM will be trained over the resulting sequences of sub-word units. BPE plays an important role in many state-of-the-art natural language modelling applications^71,72^ and provides strong baseline results on a multilingual corpus^73^. Note that the BPE is always extracted from the training data only and then applied to both the training and the test data. We follow Ref.^69^ and set the number of BPE merges to 0.4·*C* where *C* is the number of different word types observed in the training data.

On the level of words and sub-words, each unique symbol type is replaced by one unique 4-byte Unicode symbol. Each LM is then trained on the resulting sequence of Unicode symbols. On the level of characters, each LM is trained directly on the raw text.

### Sociodemographic and linguistic variables

Information on speaker population size, corpus, language family, language (identified by its ISO code), macro area, country, writing script, speaker population size, longitude and latitude are taken from Ref.^25^. EGIDS level information was initially sourced from Ref.^113^, which is reported in Glottolog^114^ (v4.2.1). Country is defined by Ethnologue as the primary country/country of origin of the language in question^15^. To ensure completeness, we manually supplemented missing data from Ref.^113^ by cross-referencing with Glottolog and Ethnologue. The EGIDS level serves as a measure of a language's endangerment status^57^. The purpose is to use the EGIDS level as a covariate to control for potential translation effects^55,111^, as languages with lower EGIDS levels could be more likely to be used as source languages, while languages with higher EGIDS levels could be more likely to be used as target languages. For example, an EGIDS level of 0 (labelled "International") pertains to the six official United Nations languages: Arabic, Chinese, English, French, Russian, and Spanish. On the other hand, languages with values of five and above pertain to languages that are not used in formal education, mass media or by the government, and they may consequently be more susceptible to (more) pronounced "translationese" influences^111^.

Additional information used in *Study 2* regarding the classification of languages into family, subfamily and sub-branch are taken from Ref.^67^. We manually added information for five languages that was missing by using publicly available genealogical classifications (ISO codes gso, lbk, lsm, npi and yan, see https://osf.io/sa9x2/ for details). Classifications in Ref.^67^ are given as comma-separated values, we define the first value as the family, the second one as the subfamily and the third one as the sub-branch e.g. for the language “Ghotuo” the classification is “Niger-Congo, Atlantic-Congo, Volta-Congo, Benue-Congo, Edoid, North-Central, Ghotuo-Uneme-Yekhee”, so the family is “Niger-Congo”, the subfamily is “Atlantic-Congo” and the sub-branch is “Volta-Congo”. Additionally, we use a phylogenetic tree provided by Ref.^82^ for the PGLS regressions and a dissimilarity matrix provided by Ref.^85^ for the SAR regressions. We take information on language range size estimates from Ref.^115^ and information on distance to water resources, altitude and two variables on climatic information (Climate PC1 and Climate PC2) from Ref.^20^. Information on the number of countries in which each language is spoken was sourced from Glottolog (v4.2.1). We manually supplemented missing data by cross-referencing with Ethnologue^83,116^. The rationale behind considering this variable as a potential covariate is to account for the varying degrees of pluricentrism^56^. For instance, languages such as Chinese or Spanish are spoken in several countries and may therefore have different codified standard forms. For further information and a discussion of potential caveats and problems regarding the assignment of environmental variables to individual languages in order to reflect local grouping structure, see Refs.^20,36^.

### Estimating LM learning difficulty

The link between understanding, prediction and compression mentioned above (“Language models”) directly implies that the better the compression, the better the language model^78^.

In *Study 1*, we take advantage of this fact by measuring the compression rate ${r}_{l}$ for different sub-sequences of increasing length *l* where ${r}_{l}$ represents the number of bits per symbols that are needed to compress the first *l* symbols. Estimating the shape of the curve of the resulting series of compression lengths gives us a measure of how well language learning succeeds^32,117^. For *PPM* as LM, we take the series of compression rates directly from Ref.^25^. Here, each document in each corpus is compressed every *m* symbols where *m* is some pre-defined corpus-specific chunk size, e.g. 1000 symbols. For the purpose of this study, we carried out a comprehensive retraining of all documents utilizing two additional language models, *PAQ* and *LSTM*_*comp*_. Due to the significant increase in training time required (see Table 1), we did not use the corpus-specific chunk size pre-defined by Ref.^25^, but compressed each document of each multilingual corpus every 5% of all symbols, resulting in 20 data points, i.e. ${r}_{l}$-values per document. Note that consistency checks revealed that *LSTM*_*comp*_ repeatedly produced inconsistent results for the following documents that belong to the United Nations Parallel Corpus^118^, see Ref.^25^ for further details: ISO code “fra”, level: words, ${r}_{l}$ at 95% and 100%; ISO code “rus”, level: characters, all ${r}_{l}$-values from 65% to 100%; ISO code “rus”, level: words, ${r}_{l}$ at 100%. Since these inconsistencies could not be resolved, we exclude these ${r}_{l}$-values in what follows.

We fit a variant of the ansatz suggested by Ref.^65^ to each series of compression rates:

1 $${r}_{l}=h+A\cdot \frac{\mathrm{log}l}{{l}^{b}}$$

where *A* > 0, *b* > 0 and $h>0$; ${r}_{l}=R({X}_{1}^{l})/l$ denotes the number of bits per symbol that are needed to compress the first *l* symbols of a document. *h* is the limiting entropy rate, *A* is a proportionality constant and *b* describes the shape of the curve and thus can be used to quantify learning difficulty as visualised in Fig. 1 and Supplementary Fig. 1. To estimate the three parameters of the ansatz function, we fit the following nonlinear function by log-least squares:

2 $${r}_{l}=\mathrm{exp}\left({h}^{*}+\mathrm{exp}({A}^{\prime})\cdot \frac{\mathrm{log}l}{{l}^{\mathrm{exp}({b}^{\prime})}}\right)+{\text{o}\!}^{\prime}_{l}$$

where ${\text{o}\!}^{\prime}_{l}$ is an independent and identically distributed (i.i.d.) error term and exp() denotes the exponential function. Since we want *A* and *b* to be positive, we set interval constraints that make sure that the optimisation algorithm will not search in the negative subspace by fitting both parameters as exponentials, i.e. we estimate ${A}^{\prime}=\mathrm{log}(A)$ and ${b}^{\prime}=\mathrm{log}\left(b\right)$. The limiting entropy rate is recovered as $h=\mathrm{exp}({h}^{*})$.

Since achieving convergence of the parameter estimates turned out to be difficult^25^, we approximate initial values in linear space, i.e., for each value of *φ* = 0.01, 0.02, …, 10, we calculate $\Phi =\frac{\mathrm{log}l}{{l}^{\varphi }}$ and fit the following linear regression by ordinary least squares:

3 $$\mathrm{log}\left({r}_{l}\right)={\beta }_{h}+{\beta }_{A}\Phi +{\text{o}\!}^{\prime}_{l}$$

where ${\text{o}\!}^{\prime}_{l}$ is an i.i.d. error term. To provide initial values to fit Eq. (2), we pick the solution of Eq. (3) where the root mean squared error is smallest and where ${\beta }_{A}>0$, then ${h}^{*}$ is initialized as ${\beta }_{h}$,${A}^{\prime}$ is initialized as $\mathrm{exp}{(\beta }_{A})$ and $b^{\prime}$ is initialized as $\mathrm{exp}({\varphi }_{m})$ where *φ*_*m*_ denotes the value of *φ* corresponding to the selected *Φ*. Further details are provided in Ref.^25^.

In *Study 2*, we compute the cross entropy *H*, i.e. the number of bits needed on average to encode/predict a training verse for each document as a function of the number of training folds as follows:

4 $${H}_{f}=\frac{R\left({T}_{f}{T}_{test}\right)-R({T}_{f})}{{N}_{v}}$$

where *f* = 1, 2, …, 9 denotes the number of folds that are used to train the *LM*,${N}_{v}$ denotes the number of verses and $R(X)$ denotes the compressed size of string *X*. ${T}_{f}$ denotes a string that consists of the concatenation of the first *f* training folds, while ${T}_{f}{T}_{test}$ represents the concatenation of ${T}_{f}$ and the test fold ${T}_{test}$. Note that on both symbolic levels (words/BPE) we also compress the mapping of unique symbols to 4-byte Unicode symbol mentioned above (“Information encoding units”) and add the resulting compressed lengths to ${R}_{f}\left({T}_{f}{T}_{test}\right)$ and ${R}_{f}({T}_{f})$.

In general, there is a strong negative correlation between cross entropy and the number of folds, for both versions, both symbolic levels, all LMs and all languages (NT version, word level—PPM2: median Pearson correlation between the log of ${H}_{f}$ and the log of *f*, *r*_med_ = − 0.90; PPM6: *r*_med_ = − 0.97; LZMA: *r*_med_ = − 0.97; PAQ: *r*_med_ = − 0.97; LSTM_comp_: *r*_med_ = − 0.98; NNCP_small_: *r*_med_ = − 0.97; NNCP_large_: *r*_med_ = − 0.98; BPE level—PPM2: *r*_med_ = − 0.73; PPM6: *r*_med_ = − 0.94; LZMA: *r*_med_ = − 0.94; PAQ: *r*_med_ = − 0.94; LSTM_comp_: *r*_med_ = − 0.98; *r*_med_ = NNCP_small_: *r*_med_ = − 0.97; NNCP_large_: *r*_med_ = − 0.98; OT version, word level—PPM2: *r*_med_ = − 0.91; PPM6: *r*_med_ = − 0.99; LZMA: *r*_med_ = − 0.99; PAQ: *r*_med_ = − 0.99; LSTM_comp_: *r*_med_ = − 0.99; NNCP_small_: *r*_med_ = − 0.98; NNCP_large_: *r*_med_ = − 0.99; BPE level—PPM2: *r*_med_ = − 0.77; PPM6: *r*_med_ = − 0.98; LZMA: *r*_med_ = − 0.99; PAQ: *r*_med_ = − 0.99; LSTM_comp_: *r*_med_ = − 0.98; NNCP_small_: *r*_med_ = − 0.98; NNCP_large_: *r*_med_ = − 0.99). This demonstrates that all LMs—on average—improve with input.

To measure language-specific learning difficulty, we fit LMERs per LM, version (NT/OT) and level (words/BPE) with the log of ${H}_{f}$ as the outcome and a fixed effect for *f*. We include (crossed) random intercepts for language and for the test fold (1–10 for PPM2, PPM6, LZMA and PAQ and, due to the significant increase in training time, 1–5 for LSTM_comp_, NNCP_small_ and NCCP_large_). In addition, we include random slopes per language, i.e. we allow the relationship between ${H}_{f}$ and *f* to be different for each language. We model the covariance structure between the random effect and the random slope for language as either (i) independent, i.e. both the effect and slope have their own variance and the covariances between effect and slope are assumed to be independent of each other or (ii) unstructured, i.e. we allow the random effect and the random slope to be correlated. In all cases, an unstructured covariance structure turned out to be better as indicated by a lower AIC. Based on the LMERs, we obtained language-specific empirical Bayes predictions or best linear unbiased predictions (BLUPs)^119^ of the random slopes, represented by variable *µ*. These BLUPs capture the interactions between *f* and language, with higher values of *µ* indicating faster learning and lower values indicating slower learning as visualized in Fig. 4. *µ* is assumed to be Gaussian with mean zero and variance $\sigma$^2^.

Models were fitted with gradient-based maximization and—since our main interest lies in the estimation of random slopes—via restricted maximum likelihood (REML) to avoid a downward-biased estimate of $\sigma$^2^, for details, see Ref.^119^.

### Statistical analyses

#### Multilevel mixed-effects linear regression (LMER)

To enhance convergence for the LMERs conducted in *Study 1* (Table 1), the outcome *b* and the fixed control variables *h* and *L* were standardized per corpus, i.e. the corpus-specific mean was subtracted from each observed value and the result was divided by the corpus-specific standard deviation. As described in the main body of the paper, our covariate candidate model set includes (i) random intercepts for corpus, language family, language, macro area, country and writing script and (ii) random slopes for corpus, language family, macro area, country and writing script. All effects are assumed to be crossed. Note, however, that—in the terminology of Ref.^120^—countries are explicitly nested within macro areas, i.e. each country occurs in exactly one macro area. In the same sense, languages are explicitly nested within language families.

To compute differences in AIC, ΔAIC, we additionally fit LMERs without a fixed effect for speaker population size. Note that in models without a fixed effect for speaker population size, we also exclude potential random slopes. We then compute ΔAIC between the full model, which includes a fixed effect and potential random slopes for speaker population size, and a reduced model that does not include a fixed effect or random slopes for speaker population size but otherwise has the same fixed and random effect structure. We counted a full model to be more apt if either its AIC value is lower than its reduced counterpart or if the fitting of the reduced model fails. In all other cases, we counted the reduced model to be better.

We model all intercepts and slopes as i.i.d. and to be independently from each other. Models were fitted with gradient-based maximization and—since our primary focus in this set of analyses is on estimating and comparing different fixed effects structures—via maximum likelihood (ML)^121–123^. We accepted any solution after a maximal number of 20 iterations. Full details on the fixed and random effect structure for each selected model are given in Supplementary Tables 1, 2. As written above, we also exclude potential random slopes in models without a fixed effect for speaker population size, since excluding the fixed effect for speaker population size while including random slopes would constrain ${\beta }_{\mathrm{LMER}}$ to be zero and thus force the random slopes to be evenly distributed around a slope of zero. To make sure that this decision does not overly influence the results, we additionally ran constrained models where we allowed for reduced models that did not include a fixed effect for speaker population size but included potential random slopes for speaker population size. We then compared AIC values between full and reduced models with the same fixed effects, random effects and random slopes. Column 6 of Supplementary Tables 1 and 2 shows that our results are also valid if such constrained models are included.

For the LMERs conducted as part of *Study 2*, our covariate candidate model set contains (crossed) random effects and slopes for macro area, country, language family, language subfamily and sub-branch. Again, countries are explicitly nested within macro areas. We also explicitly nest sub-branches within subfamilies and subfamilies within families by creating unique indicators for subfamilies/sub-branches that occur in more than one level, e.g. the sub-branch label “West” occurs in several subfamilies, e.g. “Germanic” and “Mande”. To create a unique sub-branch indicator, the corresponding sub-branches are replaced by “GermanicWest” and “MandeWest”. Further note that if there is no subfamily for a language, we also create a unique subfamily indicator within the corresponding family, e.g. for the Papuan language “Angor”, the only classification given in our data is “Senagi” for the language family. To fill in a unique group indicator for the subfamily, we use the language family. We proceed in the same way with missing sub-branches by filling in corresponding subfamilies. Analogous to Study 1, we compute ΔAIC-values. Again, we model all intercepts and slopes as i.i.d. and to be independent from each other. All models were fitted with gradient-based maximization and via ML. We accepted any solution after a maximal number of 100 iterations. Full details on the random intercept and slope structure for each selected model are given in Supplementary Table 7. Again, we tested if the inclusion of constrained models for model comparison changes the results. Columns 6 and 12 of Supplementary Table 7 show that this not the case.

#### Frequentist model averaging (FMA)

In *Study 1*, the FMA estimator is computed per LM and symbol (words/characters) for all *M* = 2430 candidate models that include a fixed effect for the log of speaker population size as^48,50,124^:

5 $${\beta }_{LMER}^{FMA}=\sum_{j=1}^{M}{\omega }_{j}{\beta }_{j}$$

where ${\omega }_{j}=\frac{{c}_{j}{\Omega }_{j}}{\sum_{i=1}^{M}{{c}_{i}\Omega }_{i}}$; ${c}_{j}$ is a binary indicator that is equal to 1 if *j* converged to a solution and 0 otherwise; ${\Omega }_{j}=exp\left(-\frac{{AIC}_{j}}{2}\right)$ where ${AIC}_{j}$ denotes the AIC value computed for *j*, likewise for *i*. Therefore, $\sum_{j=1}^{M}{\omega }_{j}=1$.

In Study 2, ${\beta }_{LMER}^{FMA}$ is computed in an analogous way per LM, symbol (words/BPE) and version (NT/OT) for the set of models consisting of *M* = 728 candidates.

#### Double-selection lasso linear regression (DS) and permutation testing

For the DS regressions, we use the log of *b* as the outcome. Our covariate of interest is the log of speaker population size. As potential control variables, we use the three different sets specified in the main part of the paper. We generate a set of variables that form third-order B-spline basis functions for both longitude and latitude each with three knots placed at the 25th, the 50th and the 75th percentiles. In addition to the log of *h*, the log of *L*, the basis functions for longitude and latitude and the set of indicator variables for the levels of corpus, language family, writing script, macro area and EGIDS, we include (log) language range, (log) distance to water resources, (log) altitude, Climate PC1, Climate PC2 and the (log) number of countries in which a language is spoken as potential controls.

The DS approach works by (i) running a lasso of speaker population size on the potential covariates, (ii) running a lasso of the log of *b* on the potential covariates. Let $\widetilde{{\text{c}}}$ denote the union of the covariates selected in (i) and (ii). As a third step, the log of *b* is regressed on the log of speaker population size and $\widetilde{{\text{c}}}$. Further information on this approach is given in Refs.^43,125^. To select the optimal value for the penalty parameter for each lasso, we use cross-validation. Standard errors are clustered at the level of individual languages, i.e. we allow for intra-language correlation. Since, as written above, the sample of languages for which we have available documents cannot be considered a random sample of the population of all languages^61,62^, we use the controls selected in step (i) and step (ii) as input for non-parametric Freedman-Lane permutation tests^63,126^. Here, we wish to test the null hypothesis that speaker population size provides no information about the outcome *b*, i.e. that the corresponding estimate coefficient is equal to zero. The procedure is as follows^127^:

1. We regress *b* (logged) against speaker population size (logged) and $\widetilde{{\text{c}}}$ and extract the observed *t*-statistic *t*_obs_ of the coefficient for speaker population size.
2. We regress *b* against $\widetilde{{\text{c}}}$ only to obtain fitted values and residuals.
3. We randomly permute the residuals and generate a new variable b* that is computed as the fitted values from step 2 and the randomly permuted residuals.
4. We regress b^*^ against speaker population size and $\widetilde{{\text{c}}}$ and extract the *t*-statistic of the coefficient for speaker population size and call that quantity t*.
5. Steps 3 and 4 are repeated 10,000 times to build the distribution of t* if the null hypothesis is true.
6. We count the number of times the absolute value of t* is at least as high as *t*_obs_ and divide the result by the number of repetitions, i.e. 10,000. The result is the permutation *p*-value.

The idea of the permutation test is that if the null hypothesis is true, we do not lose “anything essential in the data”^63^ by permuting the residuals from the reduced model (step 2), because they should not be different from the full model (step 1) and can thus be used to generate the reference distribution of the test statistic.

#### Phylogenetic generalised least squares regression (PGLS)

To account for historical relatedness among languages, we fit PGLS regressions^128^ per LM, version (NT/OT) and level (words/BPE) with learning difficulty *µ* as the outcome and speaker population size as a covariate. The PGLS approach incorporates a covariance matrix that captures the phylogenetic relatedness between languages^84^. The covariance matrix is estimated using a Brownian motion model based on a tree that represents the evolutionary relationships between different languages and is used to model the degree of similarity or dissimilarity between languages. We use a phylogenetic tree provided by Ref.^82^ that was generated using language taxonomies from Ethnologue^83^. This tree represents the evolutionary relationships among the languages in our sample and allows us to account for the non-independence of observations due to shared ancestry. Languages are identified by their ISO codes. Models are fitted by generalized least squares and estimates are derived by maximizing the log-likelihood.

To compute ΔAIC, we additionally re-fit each model without including speaker population size. As a measure of fit, we compute the coefficient of determination, *R*^2^, as the squared Pearson correlation between the observed value of *µ* and the regression model-based prediction.

#### Spatial autoregressive error regression (SAR) and permutation testing

As written above, the PGLS framework uses a single covariance matrix that represents the phylogenetic relatedness between languages^84^. That means we do not take potential non-independence due to spatial proximity into account^41^. We fit SAR regressions^45^ using a GS2SLS estimator^87^ where autocorrelated errors are treated as heteroskedastic. Individual regressions are fitted per LM, version (NT/OT) and level (words/BPE) with learning difficulty *µ* as the outcome and speaker population size as a covariate. To control for both potential sources of non-independence simultaneously, we add two spatially lagged error terms to the regression equation that are specified by the inverse of two weighting matrices. To control for spatial proximity, we compute the Haversine distance^129^ between each pair of languages based on longitudinal and latitudinal information to generate a spatial distance matrix. To control for genealogical relatedness, we used a matrix provided by Ref.^85^ that is based on word lists from the Automated Similarity Judgment Program (ASJP)^86^. Again, languages are identified by their ISO codes. To select a specific language in case there are multiple languages with the same ISO code, we select either the language whose name begins with “STANDARD_”, e.g. “STANDARD_ARABIC” or the name with the shortest length, e.g. we select “JAPANESE” over “JAPANESE_2” or “TOKYO_JAPANESE”.

To assess the significance of the estimated ${\beta }_{SAR}$-coefficients, we use the following permutation procedure:

1. We fit a SAR regression of *µ* against speaker population size (logged) and extract the observed *z*-statistic *z*_obs_ of the coefficient for speaker population size.
2. We randomly permute the speaker population size variable, re-fit the SAR model and extract the *z*-statistic of the coefficient for speaker population size and call that quantity z^*^.
3. Step 2 is repeated 10,000 times to build the distribution of z* if the null hypothesis is true (i.e. ${\beta }_{SAR}$ = 0).
4. We count the number of times the absolute value of z* is at least as high as *z*_obs_ and divide the result by the number of repetitions, i.e. 10,000. The result is the permutation *p*-value.

*R*^2^-values are computed in the same way as for the PGLS regressions.

### Supplementary Information

Supplementary Information.

## Data Availability

All parallel text data, bibliographic information on languages and the compression algorithms were taken from the sources mentioned in the “Methods” section. Data preparation, management and statistical analyses were done in Stata/MP4 (version 18.0) on a Linux server (CentOS 7.9.2009) with 756GB of available RAM. Commented Stata code plus additional R (version 4.2.2) and Python code (version 3.6.8) are available at https://osf.io/sa9x2/.
