# Supplementary material for: Languages with more speakers tend to be harder to (machine-)learn
Source: Sci Rep. 2023 Oct 28;13:18521. doi: 10.1038/s41598-023-45373-z (PMC10613286; doi:10.1038/s41598-023-45373-z)
Supplement: Supplementary file 1 — Supplementary Information. [file 41598_2023_45373_MOESM1_ESM.pdf]

Supplementary Information for

Languages with more speakers tend to be harder to (machine-)learn

Alexander Koplenig, Sascha Wolfer

Correspondence to: koplenig@ids-mannheim.de

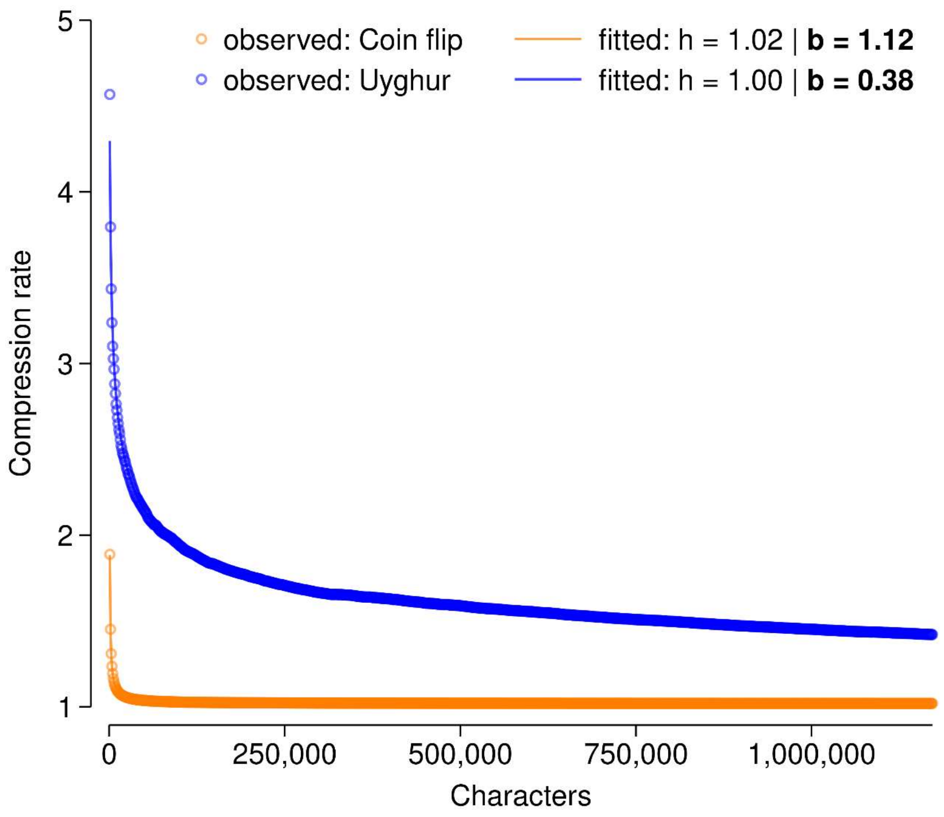

Supplementary Figure 1 | Illustration of measuring learning difficulty in Study 1. Orange circles represent observed bits-per-symbol for a synthetic dataset for a “language” with two characters (‘a’, ‘b’) that are randomly emitted (‘Coin flip’). The underlying entropy rate is therefore $h = \log_2(2) = 1.00$. Orange lines represent fitted values based on the ansatz function. As can be seen, the extrapolated entropy rate is very close to the theoretical expectation. Blue circles represent observed bits-per-symbol that are needed (on average) to encode/predict symbols based on increasing amounts of training data for a real document, here a translation of the Bible into Uyghur on the level of characters (‘uig-x-bible-romanized_parents’). The blue line represents fitted values based on the ansatz function. The extrapolated entropy rate is $h = 1.00$. The b parameter describes the shape of the fitted curves where higher values indicate fast convergence and thus lower learning difficulty. For the coin flip data, convergence is much faster, thus $b = 1.12$, whereas for the Uyghur text, the LM needs comparatively more training data and convergence to the underlying source entropy is rather slow, thus $b = 0.38$.

Supplementary Table 1 | Multilevel mixed-effects linear regression results (Study 1). Column 1: Language model. Column 2: Symbol type. Column 3: Estimated effect of speaker population size, β_{LMER}. Listed are models with the lowest AIC per LM and per symbol from a total of 2,430 models that include a fixed effect (and potential random slopes) for speaker population size. To control for the potential non-independence of data-points due to phylogenetic relatedness and geographical proximity, all models additionally include fixed covariates, random intercepts and random slopes. Standard errors and p-values are given in brackets. Column 4: Difference-in-AIC between reduced and full models. Reduced models do not include a fixed effect (and potential random slopes) for speaker population size. Column 5: Percentage of cases where the full model has a better fit (i.e. lower AIC) than the corresponding reduced model. Models that do not include a fixed effect for speaker population size but potential random slopes for speaker population size are excluded. Column 6: Percentage of cases where the full model has a better fit than the corresponding reduced model. Here, models, where $\boldsymbol{\beta}_{\textbf{LMER}}$ is constrained to be zero, are included. Column 7 – 9: selected control covariates for the model with the lowest AIC per LM and symbol. N = 3,853. Statistical significance is determined based on parametric tests. See Methods for further information on statistical methods.

<table>
<tr><th rowspan="2">LM</th><th rowspan="2">Symbol</th><th rowspan="2">$\boldsymbol{\beta}_{\textbf{LMER}}$ (s.e., p_{para})</th><th rowspan="2">ΔAIC</th><th rowspan="2">M^{+}</th><th rowspan="2">M_c^{+}</th><th colspan="3">Selected controls</th></tr>
<tr><th>Fixed effects</th><th>Random intercepts</th><th>Random slopes</th></tr>
<tr><td rowspan="2">PPM</td><td>words</td><td>-0.048 (s.e. = 0.019; p = 0.013)</td><td>58.63</td><td>100.00</td><td>99.26</td><td rowspan="6">h, L, and their interaction</td><td>corpus, macro-area, country, language family, language</td><td>corpus, macro-area</td></tr>
<tr><td>characters</td><td>-0.028 (s.e. = 0.011; p = 0.016)</td><td>98.92</td><td>100.00</td><td>99.92</td><td rowspan="5">corpus, macro-area, writing script, country, language family, language</td><td rowspan="2">corpus, macro-area, country</td></tr>
<tr><td rowspan="2">PAQ</td><td>words</td><td>-0.041 (s.e. = 0.011; p = 0.000)</td><td>46.03</td><td>100.00</td><td>98.85</td></tr>
<tr><td>characters</td><td>-0.035 (s.e. = 0.006; p = 0.000)</td><td>54.85</td><td>99.67</td><td>97.70</td><td>corpus, writing script, country, language family</td></tr>
<tr><td rowspan="2">LSTM$_{\text{comp}}$</td><td>words</td><td>-0.054 (s.e. = 0.013; p = 0.000)</td><td>55.64</td><td>100.00</td><td>100.00</td><td>corpus, macro-area, country, language family</td></tr>
<tr><td>characters</td><td>-0.022 (s.e. = 0.007; p = 0.001)</td><td>46.92</td><td>100.00</td><td>96.83</td><td>corpus, macro-area, country, language family</td></tr>
</table>

Supplementary Table 2 | Multilevel mixed-effects linear regression results (Study 1). See Supplementary Table 1 for a description of the column content. Here, we include only documents from fully parallel corpora (N = 3,224). All estimated β_{LMER}-coefficients are significant at $p < .05$, except for $\mathrm{LSTM_{comp}}$ as LM on the levels of characters, here $p = .090$.

<table>
<tr><th rowspan="2">LM</th><th rowspan="2">Symbol</th><th rowspan="2">$\boldsymbol{\beta}_{\mathbf{LMER}}$ (s.e., p_{para})</th><th rowspan="2">ΔAIC</th><th rowspan="2">$\boldsymbol{M}^{+}$</th><th rowspan="2">$\boldsymbol{M}_{c}^{+}$</th><th colspan="3">Selected controls</th></tr>
<tr><th>Fixed effects</th><th>Random intercepts</th><th>Random slopes</th></tr>
<tr><td rowspan="2">PPM</td><td>words</td><td>-0.046 (s.e. = 0.022; p = 0.034)</td><td>52.81</td><td>100.00</td><td>98.11</td><td rowspan="6">h, L, and their interaction</td><td rowspan="6">corpus, macro-area, writing script, country, language family, language</td><td>corpus, macro-area, country</td></tr>
<tr><td>characters</td><td>-0.041 (s.e. = 0.013; p = 0.002)</td><td>95.81</td><td>100.00</td><td>100.00</td><td>corpus, macro-area, country</td></tr>
<tr><td rowspan="2">PAQ</td><td>words</td><td>-0.037 (s.e. = 0.010; p = 0.000)</td><td>39.24</td><td>100.00</td><td>100.00</td><td>corpus, macro-area, writing script, language family</td></tr>
<tr><td>characters</td><td>-0.029 (s.e. = 0.007; p = 0.000)</td><td>53.42</td><td>100.00</td><td>100.00</td><td>corpus, country, language family</td></tr>
<tr><td rowspan="2">$\mathrm{LSTM_{comp}}$</td><td>words</td><td>-0.050 (s.e. = 0.018; p = 0.005)</td><td>46.18</td><td>100.00</td><td>100.00</td><td>corpus, macro-area, writing script</td></tr>
<tr><td>characters</td><td>-0.019 (s.e. = 0.011; p = 0.090)</td><td>83.50</td><td>99.84</td><td>95.84</td><td>corpus, macro-area, writing script, country</td></tr>
</table>

Supplementary Table 3 | Double-selection lasso linear regression results (Study 1). Column 1: Set of potential candidate variables to be included as controls in each model (Number of controls are given in brackets). Column 2: Language model. Column 3: Symbol type. Column 4: Number of cases. Column 5: Number of controls selected by lasso. Column 6,7: Out-of-sample R^2 of learning difficulty (column 6)/speaker population size (column 7) on the control variables selected by the lasso. Column 8: Estimated coefficient for the effect of speaker population size, β_{DS}. Robust standard errors (clustered at the level of individual languages) and p-values are given in brackets. *$p < 0.05$, ** $p < 0.01$, *** $p < 0.005$. Statistical significance is determined based on non-parametric permutation tests (see Methods for further information on statistical methods).

Candidate set	LM	Symbol	N	N_s	$R^2_{outcome}$	$R^2_{predictor}$	β_{DS} (s.e., p_{perm})
small (N_c = 225)	PPM	words	3,585	143	94.06%	79.06%	-0.004** (s.e. = 0.001, p = 0.0076)
		characters	3,585	145	93.77%	79.16%	-0.009*** (s.e. = 0.001, p = 0.0000)
	PAQ	words	3,585	155	88.90%	78.93%	-0.008*** (s.e. = 0.002, p = 0.0002)
		characters	3,585	139	89.48%	78.91%	-0.013*** (s.e. = 0.002, p = 0.0000)
	LSTMcomp	words	3,585	134	64.60%	78.66%	-0.010*** (s.e. = 0.003, p = 0.0012)
		characters	3,585	155	76.06%	78.67%	-0.009*** (s.e. = 0.002, p = 0.0000)
medium (N_c = 274)	PPM	words	3,585	164	94.01%	79.56%	-0.005*** (s.e. = 0.002, p = 0.0034)
		characters	3,585	161	93.76%	79.68%	-0.006*** (s.e. = 0.001, p = 0.0000)
	PAQ	words	3,585	174	88.99%	79.46%	-0.009*** (s.e. = 0.002, p = 0.0000)
		characters	3,585	150	89.62%	79.46%	-0.009*** (s.e. = 0.002, p = 0.0000)
	LSTMcomp	words	3,714	144	64.44%	79.33%	-0.011*** (s.e. = 0.003, p = 0.0010)
		characters	3,585	185	76.29%	79.33%	-0.008*** (s.e. = 0.003, p = 0.0036)
big (N_c = 2,226)	PPM	words	3,585	538	94.47%	77.96%	-0.002 (s.e. = 0.001, p = 0.0722)
		characters	3,585	542	93.59%	78.29%	-0.004*** (s.e. = 0.001, p = 0.0020)
	PAQ	words	3,585	433	88.44%	78.06%	-0.009*** (s.e. = 0.002, p = 0.0006)
		characters	3,714	350	89.58%	78.07%	-0.007*** (s.e. = 0.002, p = 0.0000)
	LSTMcomp	words	3,714	281	62.90%	77.95%	-0.008* (s.e. = 0.003, p = 0.0134)
		characters	3,714	482	74.54%	77.95%	-0.007** (s.e. = 0.002, p = 0.0076)

Supplementary Table 4 | Double-selection lasso linear regression results (Study 1). See Supplementary Table 3 for details. Here, only fully parallel corpora are considered.

Candidate set	LM	Symbol	N	N_s	$R^2_{outcome}$	$R^2_{predictor}$	β_{DS} (s.e., p_{perm})
small (N_c = 225)	PPM	words	3,008	146	95.10%	79.66%	-0.005*** (s.e. = 0.001, p = 0.0014)
		characters	3,008	140	95.23%	79.85%	-0.007*** (s.e. = 0.001, p = 0.0000)
	PAQ	words	3,008	138	90.72%	79.51%	-0.008*** (s.e. = 0.002, p = 0.0006)
		characters	3,008	143	91.88%	79.58%	-0.009*** (s.e. = 0.002, p = 0.0000)
	LSTMcomp	words	3,008	145	60.33%	79.43%	-0.008* (s.e. = 0.003, p = 0.0136)
		characters	3,008	144	76.61%	79.42%	-0.009*** (s.e. = 0.002, p = 0.0002)
medium (N_c = 274)	PPM	words	3,008	152	94.94%	80.29%	-0.005*** (s.e. = 0.001, p = 0.0008)
		characters	3,008	144	95.15%	80.44%	-0.006*** (s.e. = 0.001, p = 0.0000)
	PAQ	words	3,008	165	90.78%	80.11%	-0.008*** (s.e. = 0.002, p = 0.0006)
		characters	3,008	166	91.91%	80.21%	-0.006*** (s.e. = 0.002, p = 0.0008)
	LSTMcomp	words	3,008	116	51.83%	80.06%	-0.010** (s.e. = 0.003, p = 0.0072)
		characters	3,008	174	77.36%	80.04%	-0.007** (s.e. = 0.002, p = 0.0084)
big (N_c = 2,226)	PPM	words	3,008	378	95.41%	77.62%	-0.003* (s.e. = 0.001, p = 0.0214)
		characters	3,008	352	95.20%	78.15%	-0.003* (s.e. = 0.001, p = 0.0214)
	PAQ	words	3,008	464	90.84%	77.94%	-0.006* (s.e. = 0.002, p = 0.0102)
		characters	3,114	284	91.56%	77.98%	-0.005*** (s.e. = 0.002, p = 0.0034)
	LSTMcomp	words	3,008	334	58.65%	77.51%	-0.006 (s.e. = 0.003, p = 0.0718)
		characters	3,008	514	76.88%	77.69%	-0.008*** (s.e. = 0.002, p = 0.0012)

Supplementary Table 5 | Cross-fit partialing-out lasso linear regression (Study 1). Column 1: Version ('all' or only fully 'parallel'). Column 2: Set of potential candidate variables to be included as controls in each model (Number of controls are given in brackets). Column 3: Language model. Column 4: Symbol type. Column 5: Number of controls selected by lasso. Column 6: Estimated coefficient for the effect of speaker population size, β_{XPO}. Robust standard errors clustered at the level of individual languages are given in brackets. *$p < 0.05$, ** $p < 0.01$, *** $p < 0.005$. Statistical significance is determined based on parametric tests. To select the optimal value for the penalty parameter for each lasso, we use a plugin iterative formula.

Version	Candidate set	LM	Symbol	N_s	β_{XPO} (s.e., p_{para})
all (N =3,714)	small (N_c = 225)	PPM	words	67	-0.004*** (s.e. = 0.001, p = 0.001)
			characters	104	-0.011*** (s.e. = 0.001, p = 0.000)
		PAQ	words	78	-0.011*** (s.e. = 0.002, p = 0.000)
			characters	84	-0.015*** (s.e. = 0.002, p = 0.000)
		LSTMcomp	words	76	-0.011*** (s.e. = 0.002, p = 0.000)
			characters	82	-0.015*** (s.e. = 0.002, p = 0.000)
	medium (N_c = 274)	PPM	words	81	-0.005*** (s.e. = 0.001, p = 0.000)
			characters	126	-0.013*** (s.e. = 0.001, p = 0.000)
		PAQ	words	83	-0.011*** (s.e. = 0.002, p = 0.000)
			characters	94	-0.017*** (s.e. = 0.002, p = 0.000)
		LSTMcomp	words	92	-0.012*** (s.e. = 0.002, p = 0.000)
			characters	89	-0.016*** (s.e. = 0.002, p = 0.000)
	big (N_c = 2,226)	PPM	words	663	-0.004*** (s.e. = 0.001, p = 0.001)
			characters	731	-0.011*** (s.e. = 0.001, p = 0.000)
		PAQ	words	576	-0.008*** (s.e. = 0.002, p = 0.000)
			characters	567	-0.014*** (s.e. = 0.002, p = 0.000)
		LSTMcomp	words	648	-0.015*** (s.e. = 0.002, p = 0.000)
			characters	678	-0.011*** (s.e. = 0.002, p = 0.000)
parallel (N =3,114)	small (N_c = 217)	PPM	words	65	-0.007*** (s.e. = 0.001, p = 0.000)
			characters	100	-0.012*** (s.e. = 0.001, p = 0.000)
		PAQ	words	81	-0.012*** (s.e. = 0.002, p = 0.000)
			characters	82	-0.012*** (s.e. = 0.002, p = 0.000)
		LSTMcomp	words	71	-0.013*** (s.e. = 0.002, p = 0.000)

			characters	79	-0.015*** (s.e. = 0.002, p = 0.000)
	medium (N_c = 266)	PPM	words	67	-0.007*** (s.e. = 0.001, p = 0.000)
			characters	111	-0.012*** (s.e. = 0.001, p = 0.000)
		PAQ	words	88	-0.013*** (s.e. = 0.002, p = 0.000)
			characters	93	-0.012*** (s.e. = 0.002, p = 0.000)
		LSTMcomp	words	80	-0.013*** (s.e. = 0.002, p = 0.000)
			characters	81	-0.016*** (s.e. = 0.002, p = 0.000)
	big (N_c = 1,912)	PPM	words	481	-0.006*** (s.e. = 0.001, p = 0.000)
			characters	527	-0.009*** (s.e. = 0.001, p = 0.000)
		PAQ	words	529	-0.011*** (s.e. = 0.002, p = 0.000)
			characters	484	-0.012*** (s.e. = 0.002, p = 0.000)
		LSTMcomp	words	550	-0.013*** (s.e. = 0.002, p = 0.000)
			characters	518	-0.012*** (s.e. = 0.002, p = 0.000)

Supplementary Table 6 | Bayesian Linear regression results. Column 1: Version ('all' or only fully 'parallel). We first ran double-selection lasso linear regressions with the log of *b* as the outcome, the log of speaker population size as the covariate of interest and a set of potential controls that consists of the same variables as the small set described in the main part of the paper plus – since it is not possible to cluster variables at the level of individual languages for Bayesian models – indicator variables for the levels of language. The set consists of N_c = 1,505 variables for the 'all' version and N_c = 1,457 variable for the 'parallel' version. To select the optimal value for the penalty parameter for each lasso, we use a plugin iterative formula. Column 3: Language model. Column 4: Symbol type. Column 5: Number of controls selected by lasso. We then fitted Bayesian linear regressions with the log of *b* as the outcome and the log of speaker population size plus the selected controls as the covariates. We used a burn-in period of 100,000 iterations, normal priors with mean 0 and variance of 10,000 for the regression coefficients and an inverse-gamma prior with shape and scale parameters of 0.01 for the error variance. We used Gibbs sampling and simulated four chains for each model. Column 5: The Gelman–Rubin convergence diagnostic [1,2], R_c, is given in parentheses for each model. All values are below 1.2 indicating convergence. Column 6: 95% credible interval for the estimated coefficient of speaker population sized. Column 7: Estimated posterior probability that the coefficient of speaker population size is negative (in %).

**Version**	**LM**	**Symbol**	N_s	R_c	**CI$_{95\%}$**	**Test**
all (N = 3,714)	PPM	words	478	1.00	[-0.00956, -0.00475]	100.00%
		characters	394	1.01	[-0.01704, -0.01269]	100.00%
	PAQ	words	477	1.01	[-0.01667, -0.00968]	100.00%
		characters	487	1.04	[-0.02147, -0.01439]	100.00%
	LSTM$_{comp}$	words	463	1.01	[-0.01998, -0.01116]	100.00%
		characters	460	1.04	[-0.02584, -0.01663]	100.00%
parallel (N = 3,114)	PPM	words	397	1.01	[-0.01025, -0.00494]	100.00%
		characters	422	1.01	[-0.01557, -0.01102]	100.00%
	PAQ	words	405	1.03	[-0.01726, -0.00947]	100.00%
		characters	411	1.02	[-0.01644, -0.01046]	100.00%
	LSTM$_{comp}$	words	406	1.02	[-0.02610, -0.01603]	100.00%
		characters	404	1.14	[-0.02436, -0.01520]	100.00%

Supplementary Table 7 | Multilevel mixed-effects linear regression results (Study 2). Column 1: Language model. Column 2: Symbol type. The table shows random effects/slopes structure for the selected models listed in Table 4 in the main part of the paper. Columns 3-8: Results for the NT version (*N* = 504). Columns 9-14: Results for the OT version (*N* = 138). Columns 3,9: Estimated fixed effect of speaker population size, β_{LMER}. Listed are models with the lowest AIC per LM and per symbol from a total of 728 models that include a fixed effect (and potential random slopes) for speaker population size. Standard errors are given in brackets. Columns 4,10: Difference-in-AIC between reduced and full models. Columns 5,11: Percentage of cases where the full model has a better fit (i.e. lower AIC) than the corresponding reduced model (here models that do not include a fixed effect for speaker population size but potential random slopes for speaker population size are excluded). Columns 6,12: Percentage of cases where the full model has a better fit than the corresponding reduced model (here models, where β_{LMER} is constrained to be zero, are included). Column 7,8: Random effects/slopes structure for the NT version. Column 13,14: Random effects/slopes structure for the OT version.

*$p < 0.05$, ** $p < 0.01$, *** $p < 0.005$. Statistical significance is determined based on parametric tests. See Methods for further information on statistical methods.

		**Version: NT (*N* = 504)**						**Version: OT (*N* = 138)**					
**LM**	**Symbol**	β_{LMER} **(s.e.)**	**ΔAIC**	M^{+}	M_c^{+}	**Intercepts**	**Slopes**	β_{LMER} **(s.e.)**	**ΔAIC**	M^{+}	M_c^{+}	Intercepts	Slopes
PPM2	words	-0.0022*** (0.0006)	16.23	100.00	100.00	script, country, macrofamily, subfamily, subbranch	script, macrofamily, subfamily	-0.0031*** (0.0010)	11.07	100.00	100.00	macroarea, country, macrofamily, subfamily, subbranch	country, macrofamily, subfamily
	BPE	-0.0007*** (0.0002)	13.94	100.00	100.00	script, macroarea, country, macrofamily, subfamily, subbranch	script, country, macrofamily, subfamily	-0.0009*** (0.0002)	8.41	100.00	100.00	script, macroarea, country, macrofamily, subbranch	macrofamily, subbranch
PPM6	words	-0.0022*** (0.0005)	12.85	100.00	100.00	script, country, macrofamily, subfamily, subbranch	script	-0.0032*** (0.0010)	8.52	100.00	100.00	script, macroarea, country, macrofamily, subfamily, subbranch	script, macroarea, macrofamily, subfamily
	BPE	-0.0015*** (0.0003)	21.47	100.00	100.00	script, country, macrofamily, subfamily, subbranch	script, macrofamily, subfamily	-0.0019*** (0.0004)	13.29	100.00	100.00	macroarea, country, macrofamily, subfamily, subbranch	country, subfamily, subbranch
LZMA	words	-0.0021*** (0.0005)	16.00	100.00	100.00	script, macroarea, country, macrofamily, subfamily, subbranch	script, macrofamily	-0.0028*** (0.0008)	10.90	100.00	100.00	script, macroarea, country, macrofamily, subfamily, subbranch	script, country, macrofamily, subfamily, subbranch
	BPE	-0.0011*** (0.0003)	10.92	100.00	100.00	script, macroarea, country, macrofamily, subfamily, subbranch	script, macroarea, macrofamily, subfamily	-0.0013*** (0.0003)	16.13	100.00	100.00	macroarea, country, macrofamily, subbranch	macroarea, subbranch
PAQ	words	-0.0016*** (0.0005)	11.02	100.00	100.00	script, country, macrofamily, subfamily, subbranch	script, macrofamily	-0.0024*** (0.0006)	10.17	100.00	100.00	macroarea, country, macrofamily, subfamily, subbranch	macrofamily, subfamily, subbranch

	BPE	-0.0009** (0.0003)	8.01	100.00	100.00	script, macroarea, country, macrofamily, subfamily, subbranch	script, macroarea, macrofamily	-0.0010*** (0.0004)	11.63	100.00	100.00	macroarea, country, macrofamily, subbranch	macroarea, country, subbranch
LSTM comp	words	-0.0008*** (0.0003)	8.20	100.00	100.00	script, country, macrofamily, subfamily, subbranch	script, macrofamily	-0.0020*** (0.0005)	13.17	100.00	100.00	macroarea, country, macrofamily, subfamily, subbranch	macrofamily, subfamily
	BPE	-0.0012*** (0.0002)	18.89	100.00	100.00	script, macroarea, country, macrofamily, subfamily, subbranch	script, macrofamily, subfamily, subbranch	-0.0015*** (0.0003)	13.35	100.00	100.00	script, macroarea, country, macrofamily, subfamily	script, macroarea, macrofamily
NNCP small	words	0.0001 (0.0002)	0.19	33.10	50.00	script, macroarea, country, subfamily, subbranch	script, subfamily, subbranch	-0.0013*** (0.0004)	6.79	100.00	100.00	script, country, macrofamily, subfamily, subbranch	script, subfamily, subbranch
	BPE	-0.0001 (0.0003)	-0.67	9.75	50.14	script, macroarea, country, macrofamily, subfamily, subbranch	macroarea, macrofamily, subfamily	-0.0016*** (0.0003)	17.00	100.00	100.00	country, subfamily, subbranch	
NNCP large	words	-0.0005 (0.0003)	1.44	97.94	92.45	script, macroarea, country, macrofamily, subfamily, subbranch	script, macroarea, macrofamily, subfamily	-0.0015*** (0.0004)	7.99	100.00	100.00	macroarea, country, macrofamily, subfamily, subbranch	subfamily, subbranch
	BPE	-0.0007*** (0.0002)	5.01	100.00	100.00	script, macroarea, country, macrofamily, subfamily, subbranch	macroarea, macrofamily, subfamily, subbranch	-0.0017*** (0.0004)	13.43	100.00	100.00	country, subfamily	

Supplementary Table 8 | Cross-fit partialing-out lasso linear regression (Study 2). As potential control variables, we include the number of countries in which the language is spoken, third-order B-spline basis functions for both longitude and latitude each with three knots placed at the 25th, the 50th and the 75th percentiles (see Methods for details) and a set of indicator variables for the levels of writing script, macro-area, language family, language subfamily, sub-branch and EGIDS level (N_c = 417 for NT and N_c = 218 for OT). Column 1: Language model. Column 2: Symbol type. Columns 3,4: Results for the NT version. Column 3: Estimated coefficient for the effect of speaker population size, β_{XPO}. Robust standard errors are given in brackets. Column 4: Number of selected controls. Columns 5,6: Analogous results for the OT version. *$p < 0.05$, ** $p < 0.01$, *** $p < 0.005$. Statistical significance is determined based on parametric tests. To select the optimal value for the penalty parameter for each lasso, we use a plugin iterative formula.

		**Version: NT (N = 504)**		**Version: OT (N = 138)**	
**LM**	**Symbol**	β_{XPO} **(s.e.)**	N_s	β_{XPO} **(s.e.)**	N_s
PPM2	words	-0.0023*** (0.0007)	117	-0.0041*** (0.0012)	40
	BPE	-0.0006** (0.0002)	103	-0.0008* (0.0003)	49
PPM6	words	-0.0019*** (0.0006)	116	-0.0047*** (0.0011)	46
	BPE	-0.0013*** (0.0004)	108	-0.0028*** (0.0006)	51
LZMA	words	-0.0016** (0.0006)	122	-0.0035*** (0.0009)	46
	BPE	-0.0005 (0.0005)	100	-0.0017*** (0.0005)	48
PAQ	words	-0.0012* (0.0005)	124	-0.0033*** (0.0007)	48
	BPE	-0.0002 (0.0006)	104	-0.0018*** (0.0005)	46
LSTM	words	-0.0007* (0.0003)	94	-0.0025*** (0.0005)	52
	BPE	-0.0012*** (0.0003)	97	-0.0019*** (0.0005)	53
NNCPsmall	words	-0.0000 (0.0003)	100	-0.0011* (0.0005)	43
	BPE	0.0019 (0.0019)	110	-0.0016*** (0.0004)	41
NNCPlarge	words	-0.0003 (0.0003)	103	-0.0019*** (0.0006)	54
	BPE	-0.0007* (0.0003)	99	-0.0016*** (0.0005)	39

Supplementary Table 9 | Phylogenetic generalized least squares regression results (Study 2). Column 1: Language model. Column 2: Symbol type. Columns 3-5: Results for the NT version (N = 504). Columns 6-8: Results for the OT version (N = 138). Columns 3,6: Estimated effect of speaker population size, β_{PGLS}. Standard errors are given in brackets. Columns 4,7: Difference-in-AIC between reduced and full models, $\mathrm{AIC_r}$ – AIC. Columns 5,8: Coefficient of determination, R^2 (in %). *$p < 0.05$, ** $p < 0.01$, *** $p < 0.005$. Statistical significance is determined based on parametric tests. See Methods for further information on statistical methods.

		**Version: NT (N = 504)**			**Version: OT (N = 138)**		
**LM**	**Symbol**	$\boldsymbol{\beta_{PGLS}}$ **(s.e.)**	**ΔAIC**	$\boldsymbol{R^2}$	$\boldsymbol{\beta_{PGLS}}$ **(s.e.)**	**ΔAIC**	$\boldsymbol{R^2}$
PPM2	words	-0.0028*** (0.0005)	30.16	6.18	-0.0050*** (0.0010)	21.21	15.48
	BPE	-0.0007*** (0.0001)	20.62	4.39	-0.0013*** (0.0002)	25.72	18.20
PPM6	words	-0.0026*** (0.0004)	40.28	8.05	-0.0061*** (0.0010)	30.66	21.08
	BPE	-0.0018*** (0.0002)	83.37	15.58	-0.0034*** (0.0004)	59.47	35.94
LZMA	words	-0.0025*** (0.0004)	41.27	8.23	-0.0051*** (0.0008)	33.75	22.82
	BPE	-0.0015*** (0.0002)	52.31	10.22	-0.0027*** (0.0003)	52.22	32.49
PAQ	words	-0.0020*** (0.0003)	36.88	7.42	-0.0045*** (0.0007)	38.31	25.33
	BPE	-0.0013*** (0.0002)	43.40	8.61	-0.0027*** (0.0003)	49.78	31.29
$\mathrm{LSTM_{comp}}$	words	-0.0011*** (0.0002)	32.64	6.64	-0.0032*** (0.0005)	36.95	24.59
	BPE	-0.0011*** (0.0002)	38.73	7.76	-0.0021*** (0.0003)	36.47	24.33
$\mathrm{NNCP_{small}}$	words	-0.0000 (0.0002)	-1.97	0.01	-0.0017*** (0.0004)	15.23	11.74
	BPE	-0.0002 (0.0002)	-0.65	0.27	-0.0017*** (0.0003)	30.84	21.18
$\mathrm{NNCP_{large}}$	words	-0.0006*** (0.0002)	8.16	2.00	-0.0026*** (0.0005)	27.00	18.96
	BPE	-0.0008*** (0.0002)	13.90	3.10	-0.0020*** (0.0004)	26.21	18.49

Supplementary Table 10 | Spatial autoregressive error regression results (Study 2). Column 1: Language model. Column 2: Symbol type. Column 3,4: Results for the NT version (N = 414). Column 5,6: Results for the OT version (N = 126). Column 3,5: Estimated effect of speaker population size, β_{SAR}. Standard errors that are treated as heteroskedastic are given in brackets. Column 4,6: Coefficient of determination, R^2 (in %). Each model contains autoregressive error terms for phylogenetic relatedness and geographical proximity simultaneously estimated by two inverse-distance matrices. *$p < 0.05$, ** $p < 0.01$, *** $p < 0.005$. Statistical significance is determined based on non-parametric permutation tests. Note that since models are based on a GS2SLS, the computation of AIC values is not appropriate, because estimation is not based on (log) likelihood maximization. See Methods for further information.

		**Version: NT (N = 414)**		**Version: OT (N = 126)**	
**LM**	**Symbol**	**β_{SAR} (s.e.)**	**R^2**	**β_{SAR} (s.e.)**	**R^2**
PPM2	words	-0.0021*** (0.0005)	5.95	-0.0037*** (0.0011)	10.84
	BPE	-0.0008*** (0.0002)	5.19	-0.0008* (0.0003)	14.32
PPM6	words	-0.0019*** (0.0004)	8.78	-0.0036*** (0.0011)	16.69
	BPE	-0.0020*** (0.0003)	18.01	-0.0029*** (0.0005)	31.03
LZMA	words	-0.0020*** (0.0004)	9.21	-0.0027*** (0.0008)	17.15
	BPE	-0.0017*** (0.0002)	13.65	-0.0019*** (0.0004)	27.40
PAQ	words	-0.0015*** (0.0003)	8.83	-0.0025*** (0.0007)	19.74
	BPE	-0.0016*** (0.0002)	11.66	-0.0021*** (0.0004)	25.58
$LSTM_{comp}$	words	-0.0008*** (0.0002)	7.24	-0.0022*** (0.0005)	20.38
	BPE	-0.0008*** (0.0002)	8.50	-0.0016*** (0.0004)	18.89
$NNCP_{small}$	words	0.0001 (0.0002)	-0.00	-0.0014** (0.0005)	8.63
	BPE	-0.0004 (0.0002)	0.67	-0.0014*** (0.0004)	16.88
$NNCP_{large}$	words	-0.0007** (0.0002)	2.56	-0.0015** (0.0005)	14.35
	BPE	-0.0010*** (0.0002)	3.91	-0.0016* (0.0005)	13.77

References

1. Gelman, A. & Rubin, D. B. Inference from Iterative Simulation Using Multiple Sequences. *Stat. Sci.* **7**, (1992).

2. Brooks, S. P. & Gelman, A. General Methods for Monitoring Convergence of Iterative Simulations. *J. Comput. Graph. Stat.* **7**, 434–455 (1998).
